# Controlling the Crystallisation and Hydration State of Crystalline Porous Organic Salts

**DOI:** 10.1002/chem.202302420

**Published:** 2023-10-06

**Authors:** Megan O'Shaughnessy, Alex C. Padgham, Rob Clowes, Marc A. Little, Michael C. Brand, Hang Qu, Anna G. Slater, Andrew I. Cooper

**Affiliations:** ^1^ Materials Innovation Factory and Department of Chemistry University of Liverpool 51 Oxford Street Liverpool L7 3NY UK; ^2^ Leverhulme Research Centre for Functional Materials Design University of Liverpool 51 Oxford Street Liverpool L7 3NY UK

**Keywords:** crystalline porous organic salts, dehydration protocol, flow chemistry, high-throughput screening, porous materials

## Abstract

Crystalline porous organic salts (CPOS) are a subclass of molecular crystals. The low solubility of CPOS and their building blocks limits the choice of crystallisation solvents to water or polar alcohols, hindering the isolation, scale‐up, and scope of the porous material. In this work, high throughput screening was used to expand the solvent scope, resulting in the identification of a new porous salt, **CPOS‐7**, formed from tetrakis(4‐sulfophenyl)methane (TSPM) and tetrakis(4‐aminophenyl)methane (TAPM). **CPOS‐7** does not form with standard solvents for CPOS, rather a hydrated phase (**Hydrate2920**) previously reported is isolated. Initial attempts to translate the crystallisation to batch led to challenges with loss of crystallinity and **Hydrate2920** forming favorably in the presence of excess water. Using acetic acid as a dehydrating agent hindered formation of **Hydrate2920** and furthermore allowed for direct conversion to **CPOS‐7**. To allow for direct formation of **CPOS‐7** in high crystallinity flow chemistry was used for the first time to circumvent the issues found in batch. **CPOS‐7** and **Hydrate2920** were shown to have promise for water and CO_2_ capture, with **CPOS‐7** having a CO_2_ uptake of 4.3 mmol/g at 195 K, making it one of the most porous CPOS reported to date.

## Graphical abstract

**High throughput screening** was used to rapidly identify a new crystalline porous salt (CPOS). Using flow chemistry, the CPOS was scaled, and the material was shown to have permeant porosity with a carbon dioxide uptake of 4.3 mmol/g at 195 K, making it one of the most porous and scalable CPOS reported to date.

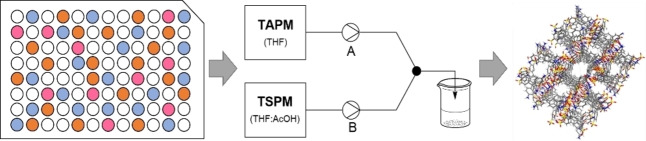

## Introduction

Crystalline porous organic salts (CPOS) are multi‐component molecular systems assembled through charge‐assisted hydrogen bonds using acids and bases.^[1]^ One of the first examples of CPOS materials are the guanidinium sulfonate (GS) series reported by Ward and co‐workers in the mid‐1990s.[^2^, ^3^] Since then, CPOS materials have found applications in areas such as protein encapsulation,[^4^, ^5^] proton conduction,[^6^, ^7^] fluorescence,[^8^, ^9^] and water harvesting.^[10]^ In principle, CPOS materials are distinguished from other classes of porous solids by their high ionic charge density, which is not found in neutral organic frameworks, such as hydrogen bonded organic frameworks (HOFs)^[11]^ and covalent organic frameworks (COFs).^[12]^ This charge density may be advantageous, for example, in applications such as water harvesting, while avoiding the use of transition metals, as used in metal–organic frameworks (MOFs).^[13]^

While a number of GS structures have been reported, the broader area of functional CPOS materials has lagged significantly behind other porous solids;[^13^, ^14^] for example, a wide range of neutral hydrogen bonded organic frameworks (HOFs) has been reported, in some cases with high levels of permanent porosity.^[15]^ In the last few years there have also been some exciting fundamental developments in CPOS area, such as the ability to tune the pores,^[16]^ the use of modulators to control crystal growth,^[17]^ and the formation of liquid and glass GS phases.^[18]^ However, there are still challenges that must be overcome to exploit the full potential of CPOS materials.

**Figure 1:**
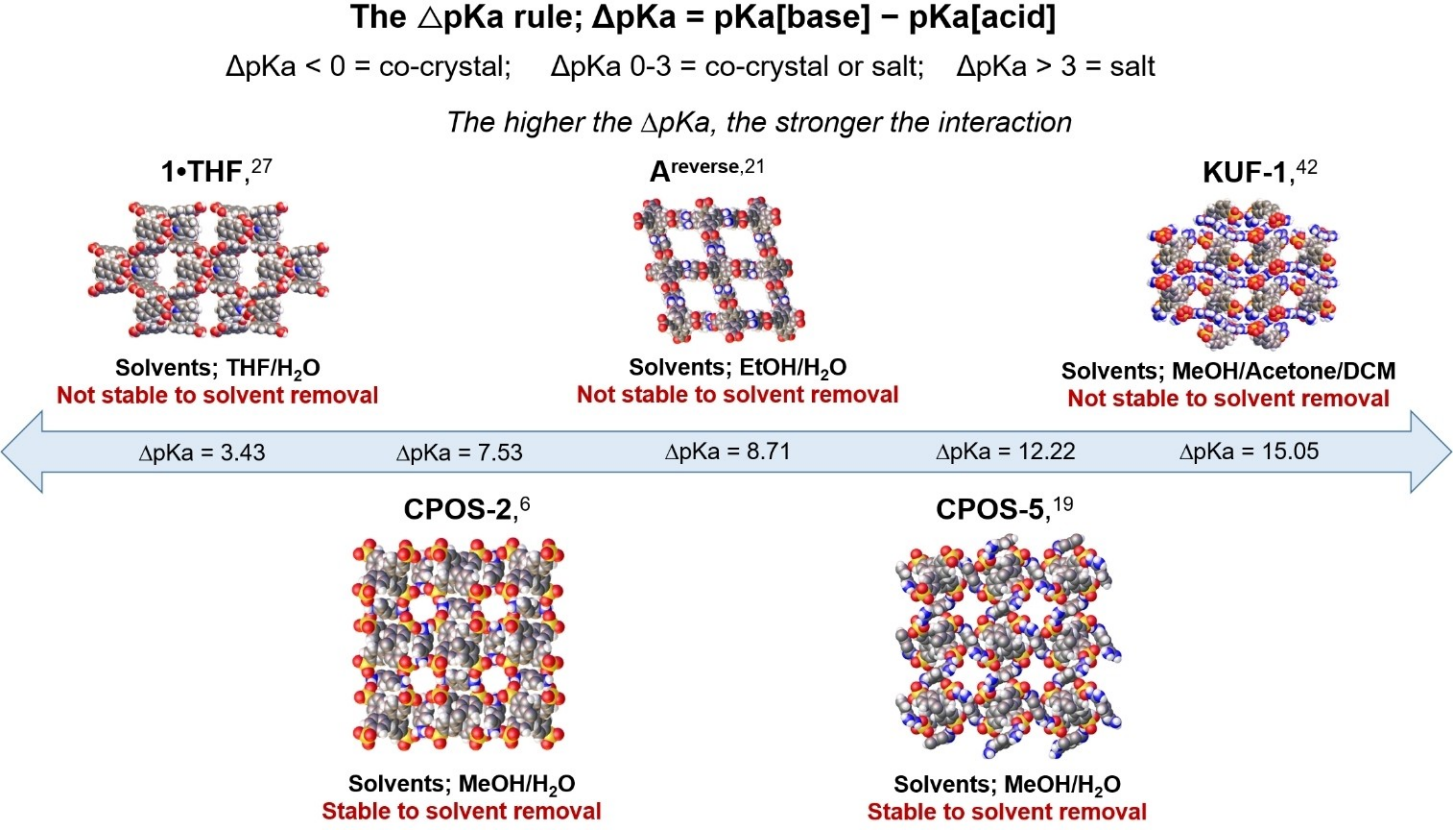
The ΔpKa rule, along with the crystal structures of some reported CPOS materials with varying ΔpKa values. The solvents used to crystallise each CPOS is reported along with their stability to removal of the solvent molecules from the voids. Image represents the challenge in forming CPOS materials with permanent porosity.

One such challenge is the choice of appropriate acids and bases to form the CPOS. Strong acids and bases give stronger ionic interactions, as demonstrated through the ΔpKa rule (Figure 1), and this typically gives a more stable framework. However, strong acids and bases may also give rise to problems including low solubility, overly high reactivity, insufficient reversibility (e. g., self‐healing during crystallization), and difficulties in activation (i. e., solvent removal) while retaining porosity.^[1]^

In 2018, Xing et al. demonstrated the importance of using a combination of strong acid and base to form stable CPOS materials when they used a combination of acids and bases with varying pKa values (Figure 1).^[6]^ To date, the best examples of CPOS materials that exhibit permanent porosity have combined sulfonic acids with amines and amidines to form relatively small pore channels.[^16^, ^19^] The most recent combination of acid and bases to form a CPOS was reported by Morshedi, et al. in 2017, using carboxylic acids and amidiniums;^[20]^ however, these structures are yet to be activated with retention of porosity.[^20^, ^21^]

The similarity in ΔpKa values of sulfonic acid/amine pairs and carboxylic acid/amidinium pairs suggests that the strength of the salt interactions alone does not dictate stability to activation. For example, CPOS in the GS series combine strong acids and bases, yet a very limited number of these materials have exhibited permanent porosity,[^22^, ^23^] despite the hundreds of crystal structures with solvent‐filled voids reported over the last 30 years.^[24]^ This illustrates the difficulties involved in forming porous CPOS's: while increased salt interaction strength can form stable structures, it may also cause problems with activation due to the strong interactions between the often‐polar crystallisation solvents and the highly charged framework. As such, the voids often collapse upon the removal of the strongly bound solvent.[^21^, ^25^] Activation can be a challenge, too, for neutral HOFs, but in general, it is much more pronounced for charged CPOS frameworks.^[26]^

To date, the crystallisation solvents for CPOS have been almost exclusively restricted to water or methanol solvent mixtures,[^1^, ^27^] based on the low solubility of the acids and bases in non‐polar/less coordinating solvents. Thus, searching for alternative solvents is one promising strategy both to aid in the activation process and to expand the range of building blocks and polymorphs achievable for CPOS frameworks. Once alternative solvents are identified, a second challenge is to produce CPOS materials at a large enough scale for function testing, and, ultimately, for the scale‐up to the desired application.^[16]^ In general, the method to control crystallisations is to slow down the rate of crystallisation; most commonly this is by using more dilute solutions, or heating to increase the solubility.

In this study, high throughput screening (HTS) was used to identify crystallisation solvents, using increased concentration but slower addition, and then promising conditions were transferred to a continuous process to targeted scalable synthesis. High throughput screening (HTS) is a method whereby automated equipment is used to rapidly test many experiments in parallel.[^28^, ^29^] HTS allows for the accelerated discovery of new materials and was first developed in the area of pharmaceuticals.^[30]^ Over the last few years, HTS has been used successfully in the area of porous material discovery, finding use in the synthesis of HOFs,^[31]^ metal–organic frameworks (MOFs),^[32]^ and porous organic cages (POCs).^[33]^

While HTS is powerful for rapid screening on small scales, it is much harder to deploy for large scale reactions.^[34]^ Flow chemistry is an attractive method for robust and scalable synthesis,^[35]^ and supramolecular chemistry is no exception.[^34^, ^36^] Flow chemistry has been used in areas of porous materials, including, MOFs, COFs, and POCs, demonstrating its potential for processing and scaling of functional materials. CPOS formation in flow has not been reported to date.

Here, we report the use of a high throughput crystallisation workflow to rapidly screen co‐crystallisation conditions of tetrakis(4‐sulfophenyl)methane (TSPM) and tetrakis(4‐aminophenyl)methane (TAPM) to form CPOS in a wide range of solvents that are typically not accessible to CPOS formation due to the substrates having poor solubility. By using a Chemspeed synthesis robot to finely control the slow mixing of the coformers, single crystal formation of a new 2D CPOS (**CPOS‐7**) was possible; a continuous flow process was then established for the synthesis of 150 mg of **CPOS‐7**, formed directly in THF with 96 % yield on a small‐scale flow apparatus. It was also found that using acetic anhydride or acetic acid allowed us to direct formation of **CPOS‐7** over a more favourable hydrate. Furthermore, this hydrate can be converted back to **CPOS‐7** using a scalable dehydration protocol. Both **Hydrate2920** and **CPOS‐7** showed promising water uptake with **CPOS‐7** also showing high CO_2_ uptake, showing the potential applications for both phases.

## Results and Discussion

**Figure 2:**
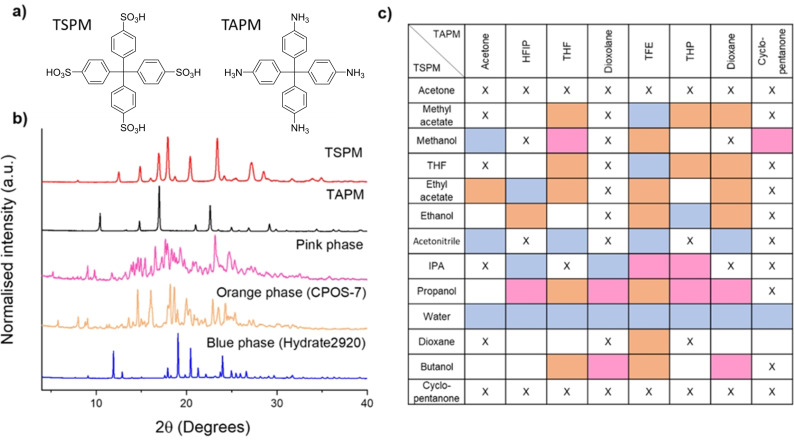
a) Chemical structures of the salt coformers, b) PXRD patterns of the individual coformers and the three main phases found during the HTS, c) crystallisation conditions used during the HTS with the three main phases highlighted: orange=**CPOS‐7**, blue=**Hydrate2920**, pink=unidentified phase, X=amorphous or oil, empty=singular or unique patterns.

First, the solubility of the two CPOS coformers (Figure 2a) was tested in 58 solvents (Table S2). Any solvents that dissolved >1 mg/mL of the coformer were classed as ‘good’ solvents. Setting the threshold solubility this low allowed us to include less polar solvents along with more typical solvents, such as water and methanol, to give a total of 104 crystallisation conditions (Figure 2c). Before starting the HTS screening for the CPOS, polymorph screening for the two substrates alone conducted under the chosen crystallisation conditions (see Supporting Information for the results). Using a Chemspeed robotic platform, the rate of addition of the TAPM to the TSPM was varied and, as expected, slower addition rates gave the best results in general (Figure S5). With the exception of water, all conditions resulted in instant precipitation upon the mixing of the solutions (Figure S6). The resulting mixtures were left aside for 7 days before the solvent was evaporated at room temperature. The samples were then transferred to a powder X‐ray diffraction (PXRD) plate and the PXRD patterns were used to cluster the materials into phases (Figure 2c). We focused on PXRD patterns that occurred over multiple conditions, since this indicated that a CPOS framework might have been formed, rather than a unique solvent‐specific solvate.

**Figure 3:**
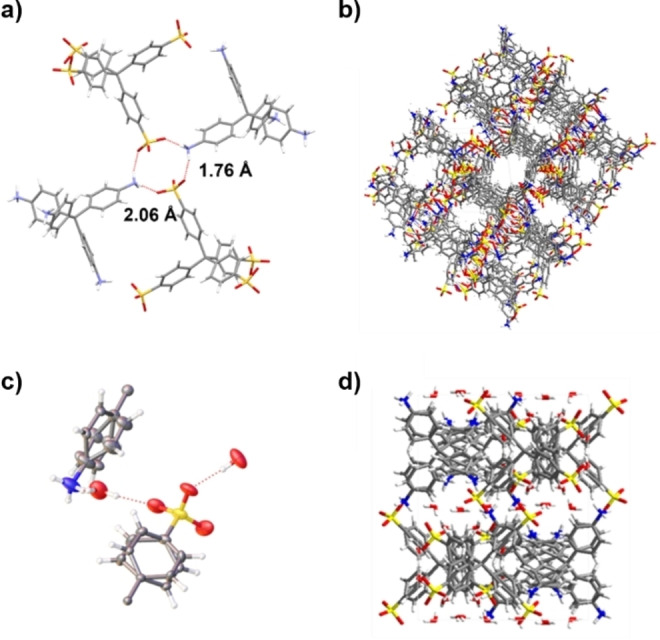
a) **CPOS‐7** with bond distances shown, b) packing of **CPOS‐7** showing voids, c) ellipsoid plot of **Hydrate2920**, d) packing of **Hydrate2920**. Note **Hydrate2920** is a separate phase, rather than a hydrated form of the **CPOS‐7** framework.

Using this HTS method, a new CPOS (**CPOS‐7**) was discovered that crystallised in the triclinic space group P1, with the asymmetric unit consisting of one molecule each of TSPM and TAPM, connected through strong charge‐assisted hydrogen bonds with distances of 2.06 Å and 1.76 Å (Figure 3a,b). **CPOS‐7** was shown to form under 19 different crystallisation conditions (orange boxes, Figure 2c). While all of these gave rise to precipitation, single crystals suitable for X‐ray diffraction were obtained under just two conditions. The most suitable crystals for diffraction formed using ethanol and dioxane; THF also gave single crystals, despite the TSPM being at saturation; in both cases, the rate of addition was 0.1 mL/min.

Highly crystalline powders of **CPOS‐7** were also obtained at a rate of addition of 0.1 mL/min under conditions where both the TSPM and TAPM were barely dissolved: for example, TSPM in methyl acetate (MeOAc) or tetrahydrofuran (THF), and TAPM in tetrahydropyran (THP) (Table S4). When the rate of mixing was set at 1 mL/min the resulting samples were largely amorphous.

In general, decreasing the rate of addition of the set at 1 mL/min the resulting samples were largely amorphous. In general, decreasing the rate of addition of the TAPM to the TSPM from 1.0 mL/min to 0.1 mL/min resulted in a drastic increase in crystallinity (Figure S5). The fact that **CPOS‐7** could be formed in a wide range of solvents, including those where the coformer solutions were saturated, demonstrates that CPOS frameworks could be formed without the need for dilute polar solutions. This opens up the possibility that other previously reported CPOS materials might be prepared in a wider range of solvents by using slow addition, which could facilitate their subsequent activation.

As demonstrated for other systems where polymorphism is important, solvent plays a vital role.^[37]^ In addition to **CPOS‐7**, a previously reported hydrated structure^[38]^ was identified, (**Hydrate2920)**, (blue phase, Figure 2c), with single crystals obtained from water solutions. **Hydrate2920** crystallised in the triclinic space group *I*4_1_/a with the asymmetric unit containing one quarter each of TSPM and TAPM, with a water molecule bridging the two molecules (Figure 3c,d). When the crystallisations were done using standard batch conditions using dilute polar solvents, the only structure found was always **Hydrate2920**, including for solvents other than water. We note that TSPM was used as a hydrate and, even after freeze drying, the material itself contained ~20 % water; moreover, none of the solvents was strictly anhydrous. It appears therefore that **Hydrate2920** forms in non‐aqueous solvents in the presence of limited quantities of water. As such, the HTS method with slow addition allowed us to identify a CPOS, **CPOS‐7** that might otherwise have been overlooked.

As **CPOS‐7** contained voids, our next aim was to test its stability to activation. We did this using gas sorption experiments, which typically require at least 50 mg of sample. **CPOS‐7** could be reliably reproduced in all solvents at 12.5 mg scales using the Chemspeed robot protocol at a rate of addition of 0.1 mL/min, and it was therefore possible to scale to 50 mg by preparing multiple batches. However, even to scale to 50 mg, the conditions were restricted to ethanol and dioxane; that is, the solvents in which the substrates were most soluble. While scaling out in this way is possible, it is somewhat laborious. We therefore focused on crystallising **CPOS‐7** in bulk using tetrahydrofuran (THF). This was more difficult due to the TSPM being at its saturation point in this solvent, which results in a more rapid rate of crystallisation. Several challenges were faced during scale up experiments, that is: (1) loss of crystallinity in **CPOS‐7** (**CPOS‐7 a**); (2) formation of **Hydrate2920**, and; (3) most commonly, formation of a mixture of **Hydrate2920** and **CPOS‐7** (Figure S12). The formation of **Hydrate2920** was likely the result of the higher water content in the solvent at larger scales; using anhydrous solvents and a nitrogen atmosphere helped to reduce this problem, but under these more inert conditions, amorphous materials were also often formed.

An advantage of neutral HOFs is that they can be easily recrystallised;^[15]^ for CPOS materials this is more difficult due to the stronger and less labile salt interactions, making recrystallisation challenging without using solvents that might be trapped in the framework. The solubility of **CPOS‐7**, **CPOS‐7 a**, **Hydrate2920** and mixtures were tested in a range of solvents (Table S2); all materials showed very poor solubility in common organic solvents. We therefore sought to find methods other than recrystallisation to overcome the issues of hydrate formation and loss of crystallinity.

The unwanted inclusion of water is a broader problem across the area of molecular crystals, not only for CPOS materials. Other than attempting to maintain strictly anhydrous conditions, there are a limited number of strategies to address this. This challenge and the general concept of hydration/dehydration is particularly relevant to materials designed for water harvesting applications. For example, using the amidinium version of TAPM, Zhang et al. reported CPOS‐6, which is a hydrated structure.^[10]^ Muang‐Non et al. made further studies on CPOS‐6,^[39]^ also reporting its high affinity for water. CPOS‐6 was shown to release water and instantly reabsorb it in humid air.^[10]^ However, more broadly, water can also disrupt crystal packing and bind irreversibly, preventing a porous phase from forming in the first place.^[40]^ Moreover, for many such hydrates, removing the water by the standard method of heating results in water being instantly reabsorbed upon cooling.^[41]^ Being able to control the strength of inclusion of water in molecular crystals and to modulate the reversibility of the process is therefore an important challenge.^[10]^

**Figure 4:**
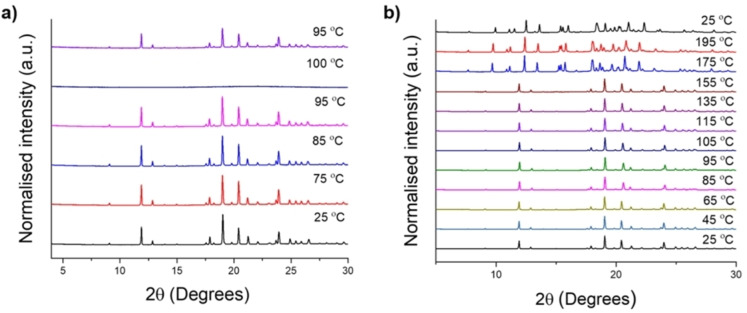
a) Variable temperature (VT) PXRD of **Hydrate2920** in a capillary in the presence of water, b) VT PXRD of an air‐dried sample of **Hydrate2920** in a capillary under a nitrogen atmosphere.

To address the hydrate problem, we used acetic anhydride and glacial acetic acid (GAA) to prevent the formation of **Hydate2920**. Acetic anhydride is a known dehydrating agent^[42]^ and was shown previously to be more effective than acetic acid by a factor of 10; however, GAA has fewer safety hazards and was used for this study. When enough acetic acid was used, the formation of **Hydrate2920** was suppressed and a pure sample of **CPOS‐7** was isolated (Figure S13), without requiring inert conditions. Even more interestingly, we found that **Hydrate2920** could be transformed into **CPOS‐7** using mild heat and GAA in the crystallisation solvent (Figure S15, Table S6). As mentioned,

**Hydrate2920** is insoluble in all common organic solvents; it only dissolves in water when heated to 100 °C, and quickly recrystallises back to the hydrate on cooling to 95 °C (Figure 4a). Typically, if a material is insoluble then this prevents recrystallisation. Here, we prevent could use GAA to both prevent the hydrate from forming and also to convert it into the desirable porous phase, **CPOS‐7**.

The affinity of **Hydrate2920** for water was tested using water isotherms. From variable temperature PXRD (Figure 4b) and differential scanning calorimetry (DSC) (Figure S10), **Hydrate2920** was shown to transform to a new unidentified phase at temperatures above 158 °C; the hydrate was therefore activated at 150 °C overnight to remove any excess water without changing the structure itself. The water isotherms showed that **Hydrate2920** adsorbed up to 12.2 mmol/g water (274 cm^3^/g) at 298 K; this is a high value for CPOS material, with only CPOS‐6 showing higher uptake (~13.8 mmol/g). We note that **Hydrate2920** is densely packed when compared to **CPOS‐7**, and hence its water uptake is quite surprising. This demonstrates that even without large voids, CPOS materials can have useful adsorption properties due to their ionic charge, as demonstrated by Kang et al. who showed that KUF‐1a absorbed NH_3_ (6.6 mmol/g) without having large pores.^[43]^

Having resolved the hydrate issue, we still faced the problem of loss of crystallinity when trying to scale **CPOS‐7** in batch. The amorphous samples of **CPOS‐7** were insoluble; again, this meant recrystallisation was not an option. However, we found that suspending the amorphous **CPOS‐7 a** in water led to **Hydrate2920**. This meant that all materials obtained, regardless of whether they were amorphous **CPOS‐7**, **Hydrate2920**, or mixtures thereof, could be transformed to the desired **CPOS‐7** framework by converting to **Hydrate2920** and then dehydrating using GAA. Once formed, in the anhydrous state **CPOS‐7** was found to be more stable toward hydration; samples stored in a capped vial for more 18 months showing no PXRD peaks for **Hydrate2920**, although heating in water gave **Hydrate2920**. To our knowledge, this is the first demonstration of controlling the formation of hydrates followed by chemical dehydration to form a porous CPOS. This method allowed for 100 % conversion of the starting coformers into the desired **CPOS‐7**, with the reconstruction from **Hydrate2920** being reproducible on scales of up to 150 mg, which was the largest scale tried – more than enough for gas sorption measurements. Even though we had now found a reproducible method to form **CPOS‐7** on large scales we still wanted to optimise the crystallisation of **CPOS‐7** directly in non‐polar solvents. Therefore, we decided to explore flow chemistry for the scaling of the crystallisation of **CPOS‐7** since the benefits offered by strict control over stoichiometry and mixing make it an attractive option.

The best conditions from the HTS were used as a starting point: ethanol/dioxane (80 °C) and saturated THF (20 °C). A Harvard Apparatus dual syringe pump was used to deliver the two coformer solutions to a Y‐piece where they were mixed – for ethanol/dioxane, the mixing piece and tubing were heated to 80 °C and the coformers were pumped at 0.5 mL/min each; with THF, the reaction was conducted at room temperature at the same flow rate and residence time. There was little difference between the two solvent systems: both gave the hydrated form of the salt (**Hydrate2920**), but the easier removal of THF meant that it was preferred. If the product was dried too quickly, only amorphous material was obtained, hence, the product was collected as a single batch and the solvent allowed to evaporate slowly. On larger scales, the excess solvent could be carefully decanted to speed up this process.

The effects of residence time and flow rate on the quality of the salt formation were investigated for the THF system. The flow rate was varied from 0.1 mL/min to 1.0 mL/min total flow rate, keeping the same overall setup (Figure S24). The next hurdle was to directly form **CPOS‐7** under continuous flow conditions, avoiding the formation of **Hydrate2920**. As for batch processes, it was found that acetic acid was required as a co‐solvent for the TSPM solution (up to 7 % v/v), as well as freshly freeze‐dried TSPM, in order for the reaction to preferentially form the porous salt. To maximise throughput, we focussed on optimising the conditions for the total flow rate of 1.0 mL/min.

Having found optimal conditions for the formation of the salt using a syringe pump, the reaction was transitioned to the fully continuous platform of a Vapourtec E‐series flow reactor, whereby peristaltic pumps could allow for uninterrupted production of salt. Using the same concentrations, the reagents were pumped at 0.5 mL/min each with a residence time of 35 seconds (Figure 6a), and 150 mg of **CPOS‐7** was obtained in one continuous process at 45 mg/h; the scale was only limited by the availability of the starting materials, and theoretically larger scales could be delivered via longer experiment times. In batch, scaling out the same quantity would take more than double the time than in flow. PXRD showed that this material had significantly higher levels of crystallinity compared to batch‐prepared material in THF, matching that of the most optimised batch conditions of EtOH/dioxane (Figure 6b).

**Figure 5:**
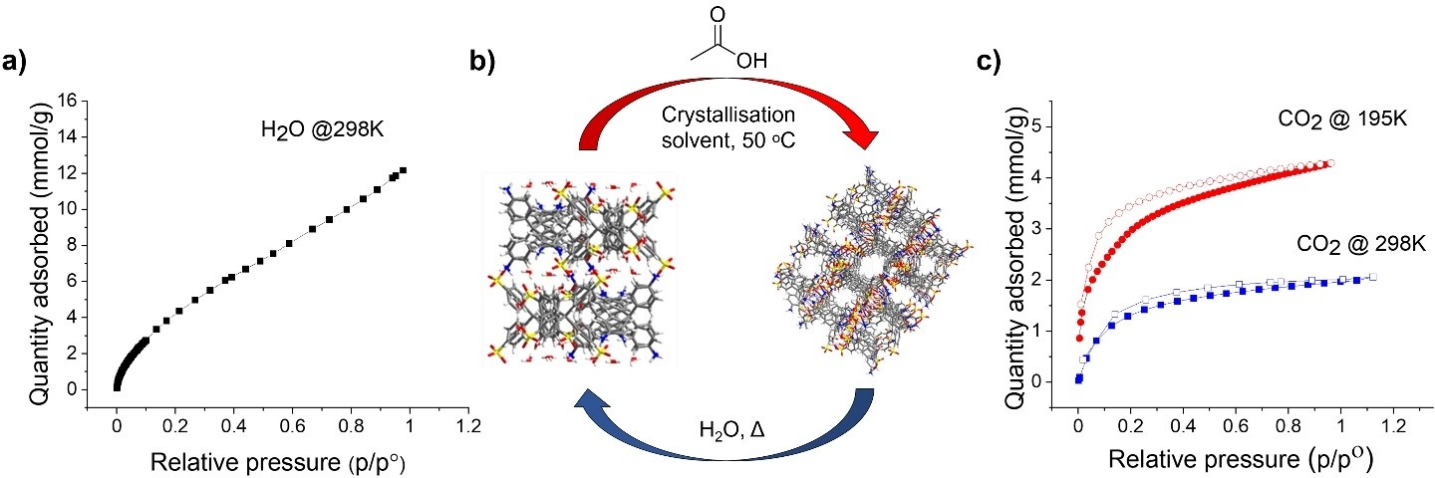
a) Water isotherm for **Hydrate2920**, b) illustration showing the transformation between the densely packed **Hydrate2920** structure and **CPOS‐7**, c) gas isotherm for **CPOS‐7**, filled symbols for adsorption and empty for desorption.

Obtaining such an increased level of crystallinity and scaling using one of the most difficult crystallisation conditions for **CPOS‐7**, shows the promise of using flow chemistry to control the crystallisation of CPOS materials. With such a positive result of **CPOS‐7** using flow, it is very plausible that this method would show the same if not better results for other CPOS materials which can be made easily at small scales in batch. The reaction was typically performed at equal flow rates with a 1 : 1 stoichiometry of the two coformers. However, preliminary result showed that doubling the concentration of the more soluble TAPM and halving the flow rate of the TAPM solution could also reliably form **CPOS‐7** (Figure S28), indicating that the throughput of the reaction might be optimised further. **CPOS‐7** was shown to have good thermal stability and could therefore be activated at 150 °C under dynamic vacuum overnight. The material made from the reconstruction of **Hydrate2920** was shown to have a maximum CO_2_ uptake of 4.3 mmol/g (96 cm^3^/g) at 195 K (Figure 5c), making it one of the more porous salts reported to date (Table 1). The CO_2_ uptake of **CPOS‐7** is slightly higher than **CPOS‐5** (88 cm^3^/g at 195 K),^[19]^ and a porous salt with unique molecular rotor dynamics reported by Bracco et al.^[45]^ *(*86 cm^3^/g at 195 K); it has greater CO_2_ uptake than other reported CPOS.[^6^, ^46^] The only CPOS materials with higher CO_2_ uptakes are **TPMA/MTBPS** (111 cm^3^/g @195 K), **TPMA‐F/MTBPS** (182 cm^3^/g@195 K) and **TPMA‐Br/MTBPS** (115 cm^3^/g @195 K).^[16]^ See Table 1 for more comparisons between CO_2_ uptakes between **CPOS‐7** and selected previously reported systems.

**Figure 6:**
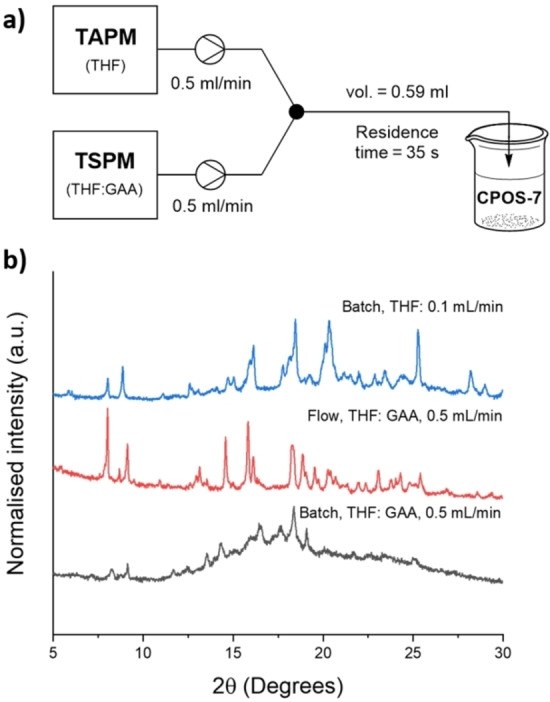
a) Flow reactor schematic for the continuous synthesis of **CPOS‐7**. 1 mg/mL stock solutions of TSPM and TAPM were used to obtain up to 150 mg of **CPOS‐7**, b) PXRD comparison of **CPOS‐7** scaled in similar conditions in batch (black line) and flow (red line), and the PXRD for **CPOS‐7** in batch when decreasing the rate of addition, which gave the best result in batch using THF.

**Table 1:** Highlighting the advantages of using HTS and flow chemistry to identify and scale CPOS.

Name	CO_2_ uptake [cm^3^/g]	Mass [mg]	Crystallisation solvents	Activation conditions
TPMA/ MTBPS^16^	111^[a]^	2	1,2,4‐TCB/DMF	SCFCO_2_
TPMA−F/ MTBPS1^16^	182^[a]^	2	Mesitylene/MeOH	SCFCO_2_
TPMA−Cl/ MTBPS1^16^	115^[a]^	2	Benzonitrile/DMA	Solvent swap
TPMA−Br/ MTBPS^16^	82^[a]^	2	Benzonitrile/DMF	Solvent swap
CPOS‐1^6^	~83^[a]^	52	MeOH	150 °C
CPOS‐2^6^	27^[b]^	29	H_2_O/MeOH	150 °C
CPOS‐5^19^	~88^[a]^	32	NaOH/H_2_O/THF	150 °C
CPOS‐6^10^	9^[b]^	18	MeOH/water	N/A
CPOS‐7	96^[a]^	150	THF/GAA	150 °C
HOF‐GS‐10^23^	42^[a]^	31	MeOH/Acetone	Solvent swap
*d*‐POS‐1^43^	87^[a]^	N/A	MeOH/o‐chlorotoluene	150 °C

Measurement temperatures: [a] 195 K. [b] 273 K.

The CO_2_ uptake of **CPOS‐7** made in flow was also tested which showed a slightly lower (~10 %) CO_2_ uptake compared to batch (3.8 mmol/g compared to 4.3 mmol/g; Figure S30), although the synthesis through conversion of **Hydrate2920** using GAA had undergone significantly more optimization. Given the simplicity of obtaining large batches using flow, further optimization for gas uptake as well as scalability could easily be performed in flow.

The water uptake of **CPOS‐7** was also tested, with the results being very similar to **Hydrate2920**. **CPOS‐7** showed a water uptake of 10.3 mmol/g (231 cm^3^/g) at 298 K (Figure S21), which is above the water uptake for several other CPOS materials. PXRD analysis taken before and after the water sorption measurements show no indication of **Hydrate2920** forming (Figure S22), providing further evidence of its resilience to water once formed as a consequence of the strong salt interactions. Only when heating in water for over 24 h will **CPOS‐7** transform to the hydrate phase.

## Conclusions

In summary, we have used HTS and flow chemistry for the first time to control the rapid crystallisation of CPOS. Using HTS with control of mixing a new CPOS (**CPOS‐7**) was discovered and shown to form in saturated solutions; therefore, allowing the use of less polar solvents than the water/alcohol solvents that are often reported Using flow chemistry **CPOS‐7** could be reliably scaled directly in THF. Most importantly these results show for the first time that CPOS can be formed in high crystallinity in a range of solvents for which the coformers have low solubility. We believe this has a number of impacts for this area; from the ability to discover polymorphs through the expansion of solvents used, to the expansion of the range of acids and bases which may have lower solubility. Most importantly, by avoiding highly polar solvents in the first place the issue of collapse of the CPOS may be avoided. Not only does this work have implications for CPOS, but solubility of molecules can cause problems across the general area of molecular crystals. **CPOS‐7** was shown to be sensitive to hydration, but for the first time for CPOS we have shown the ability to avoid the hydrate by simple addition of a dehydrating agent to the crystallisations. Further, while the hydrate was insoluble, as was any amorphous material, it could be reconstructed back to **CPOS‐7** in a facile manner. This allowed for simple and quick conversion between two polymorphs, each with their own advantages (Figure 5): **Hydrate2920** showed high water uptake and stability, while **CPOS‐7** showed high stability and a maximum CO_2_ uptake of 4.3 mmol/g making it one of the most porous CPOS materials reported. We believe this dehydration process not only has an implication for CPOS but may prove useful for the wider area of crystal engineering. These techniques have allowed for a highly stable CPOS to be formed directly on larger scales than other CPOS (Table 1) and for amorphous or hydrated material forms to be converted to porous forms without compromising the stability.

## Supporting Information

Additional references cited within the Supporting Information.[^47^, ^48^, ^49^, ^50^, ^51^, ^52^]

Deposition Numbers 2267601 (for **CPOS‐7**), 2267602 (for **Hydrate2920**), 2267604 (for **TSPM_19**), 2267605 (for **TSPM_37**) and 2267603 (for **TAPM_2610**) contain the supplementary crystallographic data for this paper. These data are provided free of charge by the joint Cambridge Crystallographic Data Centre and Fachinformationszentrum Karlsruhe Access Structures service.

## Conflict of interest

The authors declare no conflict of interest.

1

## Supporting information

As a service to our authors and readers, this journal provides supporting information supplied by the authors. Such materials are peer reviewed and may be re‐organized for online delivery, but are not copy‐edited or typeset. Technical support issues arising from supporting information (other than missing files) should be addressed to the authors.

Supporting Information

## Data Availability

The data that support the findings of this study are available in the Supporting Information of this article.
