# Supplementary material for: Controlling the Crystallisation and Hydration State of Crystalline Porous Organic Salts
Source: Chemistry. 2023 Oct 6;29(64):e202302420. doi: 10.1002/chem.202302420 (PMC10946969; doi:10.1002/chem.202302420)
Supplement: Supplementary file 1 — Supporting Information [file CHEM-29-0-s001.pdf]

Chemistry–A European Journal

Supporting Information

Controlling the Crystallisation and Hydration State of Crystalline Porous Organic Salts

Megan O'Shaughnessy,* Alex C. Padgham, Rob Clowes, Marc A. Little, Michael C. Brand, Hang Qu, Anna G. Slater,* and Andrew I. Cooper*

Materials and Methods

Materials: Tetraphenylmethane was purchased from Manchester Organics. All other chemicals were purchased from Sigma-Aldrich and used as received, except for chlorosulfonic acid which was distilled prior to use.

NMR: ^1H and ^{13}C NMR spectra were recorded at 400 MHz on a Bruker Advance 400 NMR spectrometer. Chemical shifts are reported in ppm with reference to internal residual protonated species of the deuterated solvents used for ^1H and ^{13}C analysis.

Thermogravimetric Analysis (TGA). Thermogravimetric analysis was carried out using a Q5000IR (TA Instruments) with an automated vertical overhead thermobalance. Samples (3 – 5 mg) were heated in platinum pans at a rate of 10 °C/min unless stated otherwise.

Differential Scanning Calorimetry (DCS). DSC measurements were conducted on a TA Q2000 (instrument with a Refrigerated Cooling System 90 and an autosampler) at 10 ^{o}C/min under a dry N_2 atmosphere.

Powder X-ray Diffraction (PXRD): Powder X-ray diffraction data were collected in transition mode on powder samples held on thin Mylar film in aluminium well plates on a Panalytical Empyrean diffractometer equipped with a high throughput screening XYZ stage, X-ray focusing mirror, and PIXcel detector, using Cu-Kα (λ = 1.541 Å) radiation.

Single Crystal X-ray Crystallography: Single crystal X-ray data were measured on a Rigaku MicroMax-007 HF rotating anode diffractometer (Mo-Kα radiation, λ = 0.71073 Ã, Kappa 4-circle goniometer, Rigaku Saturn724+ detector). Rigaku frames were converted to Bruker compatible frames using the programme ECLIPSE[1]. Absorption corrections, using the multi-scan method, were performed with the programme SADABS[2]. The structure was solved with SHELXT,[3] and refined by full-matrix least-least square on $[F]^2$ SHELXL,[4] interfaced through the program OLEX2.[5] H atom positions for the C-H groups were refined using the riding model. For full refinement details, see supporting CIF files.

Gas Sorption Analysis: Nitrogen isotherms were collected at 77K using an ASAP2420 volumetric adsorption analyser (micrometrics instruments Corporation). Carbon dioxide isotherms were collected up to a pressure of 1200 mbar on a micrometrics ASAP2020 volumetric adsorption analyser at 273K. Carbon dioxide isotherm at 195K and water isotherms at 298K were collected using Micromeritics 3flex volumetric adsorption analyser.

High throughput crystallisation screens: High-throughput crystallisation screens were carried out on a Chemspeed SWING POWDERDOSE robotic platform equipped with both solid and liquid dispensing tools (**Figure S1**). Liquid handling was carried out via a four-needle overhead dispensing tool powered by 4 syringe pumps (syringe volume 10 mL). After each dispense, the syringe was washed with ethanol and dried before the next solvent was dispensed. HT crystallisations were carried out in standard 8 ml glass vials, held in removable racks (80 vials in a 16x5 array). The dispensing was carried out at RT in a closed hood system.

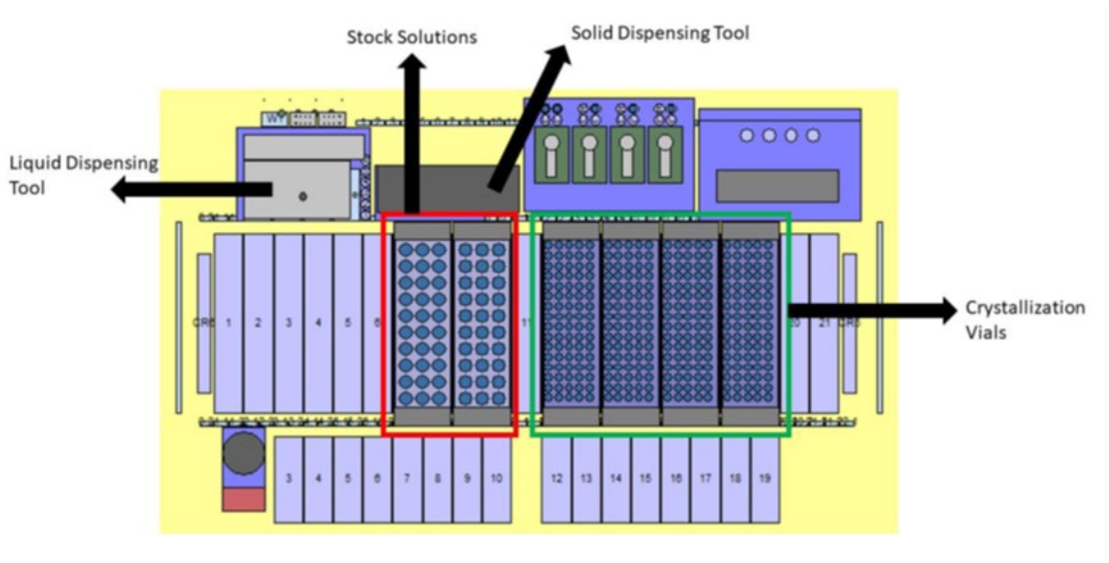

Figure S1. SWING POWDERDOSE robotic platform setup for HT crystallisation experiments.

Synthesis of TSPM

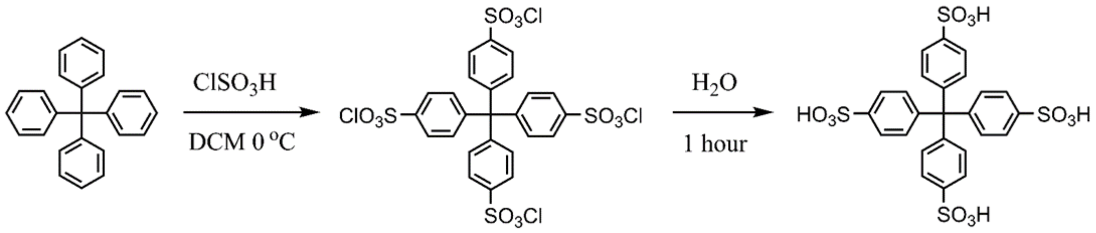

A modification of the procedure of Nangia *et al*[4] was used for this reaction. Tetraphenylmethane (8.28 g, 25.84 mmol) was sonicated in dry CH_2Cl_2 under an inert N_2 atmosphere, freshly distilled chlorosulfonic acid (8.7 mL, 130.7 mmol. 4 eq) was added dropwise over 1 hour with stirring at 0 ^oC and left for 1 hour before another 4 eq of freshly distilled chlorosulfonic acid was added in the same manner as before. In total 24 eq of chlorosulfonic acid was added in the same manner. Once the reaction had run to completion the thick brown suspension was filtered off and washed thoroughly with CH_2Cl_2. Distilled water (30 mL) was added dropwise to the solid at 0 ^oC and the resulting mixture was left stirring for 3 hours. The brown solution was freeze-dried and the resulting solid was washed

thoroughly using CH_2Cl_2 to give the final product as a pale brown solid. The solid was further washed with CH_2Cl_2 and cold water (10 mL). (9.9 g, 60% yield). ^{1}H NMR (400 MHz, D_2O, δ (ppm)): 7.48 (d, J = 8 Hz, 8H), 7.13 (d, *J* = 8 Hz, 8H). MS (ES-); 389.9769 (100 %, $[M-H]^-$). Data in accordance with literature values.[4]

Table S1. Table demonstrating the different potential acids and bases used to form CPOSs.

**Acid (pKa)**	**Base (pKa)**	**ΔpKa**	**Notes**
**-1.45**<br>**TSPM**<br>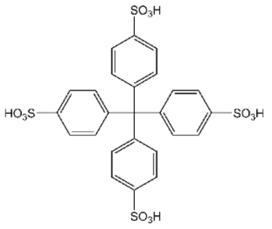	**5.75**<br>**TAPM**<br>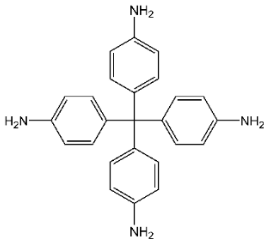	**7.2**	**CPOS-7 (this work)**<br>Solvents; large range of solvents, see Figure 2c.<br>Stable to solvent removal
**-1.45**<br>**TSPM**	**6.08**<br>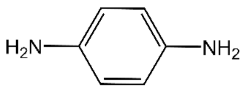	**7.53**	**CPOS-2**[,6]<br>Solvents; H_2O/MeOH<br>Stable to solvent removal
**-1.45**<br>**TSPM**	**10.77**<br>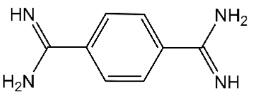	**12.22**	**CPOS-5**[,19]<br>Solvents; NaOH/H_20/THF<br>Stable to solvent removal
**-1.45**<br>**TSPM**	**13.6**<br>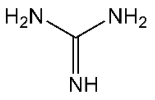	**15.05**	**KUF-1**[,43]<br>Solvents; MeOH/acetone/DCM<br>Not stable to solvent removal
**3.45**<br>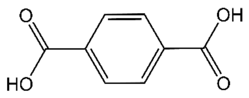	**12.20**<br>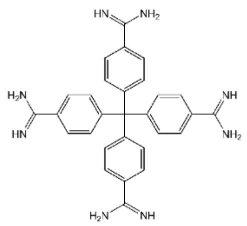	**8.71**	**A**[reverse,21]<br>Solvents; EtOH/H_20<br>Not stable to solvent removal

Solubility testing of TSPM and TAPM and sample labelling

58 solvents were used to test the solubility of both TSPM and TAPM, these solvents can be seen in **Table S2**. The general procedure for the solubility testing was as follows; 0.5 mL of solvent was added

to a vial containing 15 mg of TSPM or TAPM. The sample was then agitated by stirring and if the sample didn’t dissolve another 0.5 mL of solvent was added, again the sample was agitated; this process was repeated until the sample either dissolved or a molar concentration of 1 mg/mL had been reached.

Table S2. The solvents used during the solubility screening of TSPM and TAPM.

Diethyl ether	Pentane	DCM	Acetone	Methyl acetate
1,1,1,3,3,3-Hexafluoro-2-propanol	Chloroform	Methanol	THF	*n*-hexane
Diisopropyl -ether	1,3-Dioxolane	Ethyl acetate	Trifluoroethanol	Ethanol
Cyclohexane	Acetonitrile	2-Propanol	2,2-Dimethoxy propane	Tetrahydropyran
1-Propanol	*n*-heptane	Water	1.4-Dioxane	1,4-Difluorobenzene
Hexafluoro-benzene	1,3-Dioxane	emim [Tf_2N]	1-Butanol	Tetrachloro-ethylene
Cyclopentanone	Chlorobenzene	Toluene	Triethyl orthoformate	*N, N*-dimethyl formamide
Cyclohexanone	*m*-xylene	*p*-xylene	*o*-xylene	Dimethyl acetamide
N, N-diethyl formamide	DMSO	Ethylene glycol	Octanol	1-Methyl-2-pyrrolidinone
Mesitylene	*Y*-Butyrolactone	Butyl benzene	1,2-Dimethoxy benzene	1,3-Dimethoxy benzene
1,3-Propanediol	Diphenyl ether	bmim [BF_4]	Anisole	Tetradecane
Methyl-tetrahydrofuran	*tert*-butyl methyl ether	*N*-methyl-morpholine		

The solvents that gave a solubility of between 1-5 mg/mL were considered good solvents while any solvent that did not dissolve the sample at 1 mg/mL were considered bad solvents. Due to the known issue of forming solvates and the difficulty in removing coordinating or high boiling point solvents from any potential CPOS the number of solvents chosen from the possible options was limited to low boiling points and/or low coordinating solvents. The solvents used in the high throughput screening of the CPOS can be seen in **Table S3**, along with their sample code and the co-former they dissolved.

The crystallisation samples for the HT polymorph screen were coded in the following way; Co-former_ (good solvent for co-former)(good solvent for corresponding co-former).

For example, for polymorph sample TAPM dissolved in its good solvent acetone (solvent no. 10), mixed with TSPM's good solvent, methanol (solvent no. 14), the code was; TAPM_1014.

To give a second example, for polymorph sample TSPM dissolved in its good solvent Methyl acetate (sample no. 11) mixed with TAPM's good solvent THF (sample no. 15), the code was; TSPM_1115

Table S3. The chosen solvents to use during the HT salt crystallisation screens with the co-former they dissolved.

10. Acetone (TSPM + TAPM)	11.Methyl acetate (TSPM)	12. 1,1,1,3,3,3-Hexafluoro-2-propanol (TAPM)	14. Methanol (TSPM)
15. Tetrahydrofuran (TSPM + TAPM)	18. 1.3-Dioxolane (TAPM)	19. Ethyl acetate (TSPM)	20. Trifluoroethanol (TAPM)
21. Ethanol (TSPM)	23. Acetonitrile (TSPM)	24. 2-Propanol (TSPM)	26. Tetrahydropyran (TAPM)
27. 1-propanol (TSPM)	29. Water (TSPM)	30. 1,4-Dioxane (TSPM + TAPM)	35. 1-Butanol (TSPM)
37. Cyclopentanone (TSPM + TAPM)			

Polymorph screening of TSPM and TAPM

Polymorph screening of TSPM and TAPM was carried out in the chosen solvent systems for the co-crystallisations before the high throughput screening of the CPOS. This was to create a library of PXRD data for the individual co-formers that would allow a more rapid high throughput analysis of PXRD data for the co-crystallisations. The general procedure for the polymorph screening consisted of dissolving 15 mg of TSPM/TAPM in their good solvent before adding the desired volume of the corresponding good solvents for the other co-former. The solvent was then left to evaporate at room temperature before the resulting powder or single crystals were transferred to a PXRD plate and analysed. The PXRD plate containing the polymorphs was placed under vacuum at variable temperatures in-between which PXRD data was recorded until it was deemed likely all the solvent had been removed.

Alongside the previously reported polymorph of TSPM by Nangia *et al.*[4] two new solvate were discovered during this work (TSPM_19 and TSPM_37). Single crystals of **TSPM_19** were grown by leaving a sample of TSPM in EtOAc (5 mg in 5 mL) for a week undisturbed. For a displacement ellipsoid plot of **TSPM_19** see **Figure S2a,** CCDC: 2267604**.** Single crystals of **TSPM_37** were grown by leaving a solution of TSPM in cyclopentanone (5 mg in 1 mL) for a week undisturbed. For a displacement ellipsoid plot of the asymmetric unit see **Figure S2b,** CCDC**:** 2267605. See **Figure S3** for the PXRD patterns of the different TSPM polymorphs.

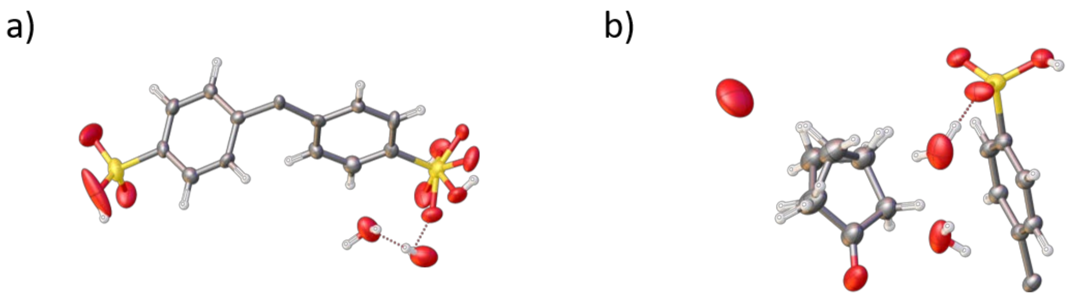

TSPM_19
a = 16.592(4) Å, b = 7.3530(17) Å,
c = 16.844(4) Å, β = 97.743(6) °,
V = 2036.3(8) Å^3

TSPM_37
a = 19.2390(14) Å, b = 19.2390(14) Å,
c = 7.6186(5) Å, α,β,γ, = 90.
V = 2819.9(5) Å^3

Figure S2 a) Displacement ellipsoid pot off the asymmetric crystal structure of **TSPM_19** from ethyl acetate, b) Displacement ellipsoid plot of the asymmetric unit cell of **TSPM_37** from cyclopentanone.

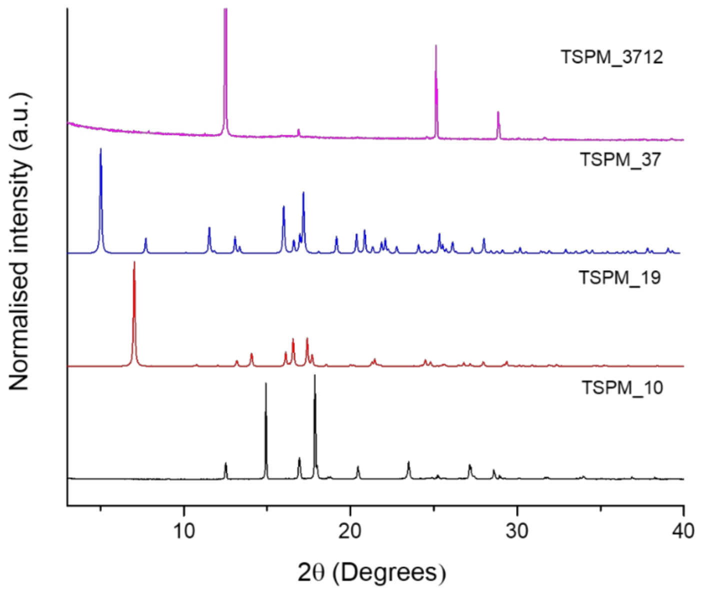

Figure S3. PXRD patterns for the different polymorphs formed during the polymorph screening of TSPM.

It was found the most thermodynamically stable polymorph of TAPM (**TAPM_1029**) is when it crystallises out in space group $I4_1/\alpha$ - this polymorph had been previously reported by J.D. West *et al.*[11] This is the only polymorph of TAPM reported in literature. Alongside the previously reported polymorph, a new single crystal structure of TAPM (**TAPM_2610**) was found (**Figure S4a**), CCDC: 2267603, along with the indication via PXRD of another phase (**TAPM_1015**) (**Figure S4b**).

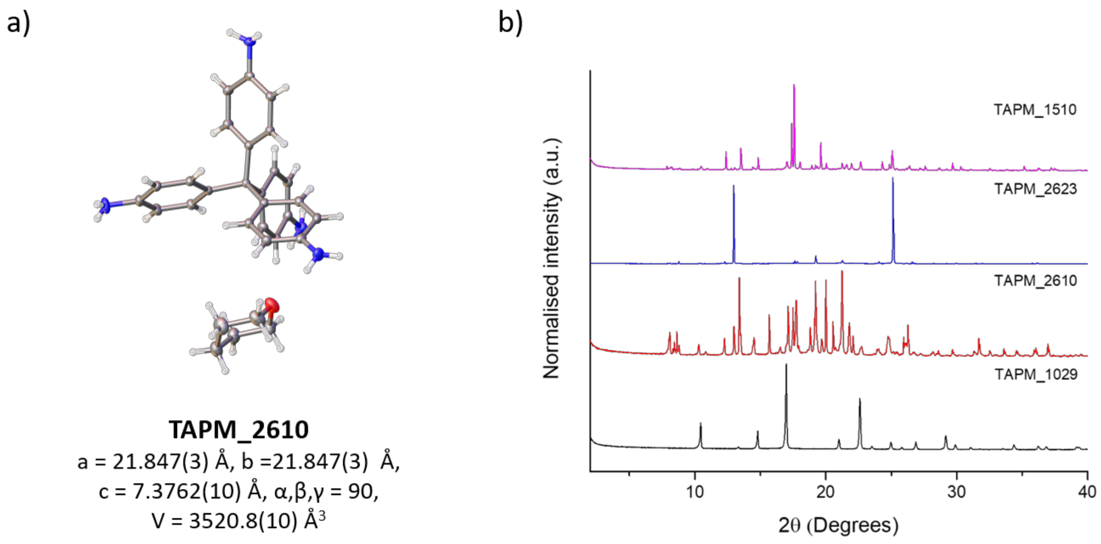

Figure S4. a) Displacement ellipsoid pot off the asymmetric crystal structure of TAPM_2610 grown from tetrahydropyran and acetone, b) shows the PXRD patterns for the different phases of TAPM identified during the screening.

HT CPOS screening

For the salt crystallisation screen stock solutions of TSPM and TAPM were prepared in the good solvents at room temperature, details of the concentrations and amounts added can be seen in **Table S4**. For all the screens on the Chemspeed, the crystallisations were left undisturbed for 7 days before being checked for single crystals. All samples that gave single crystals were analysed via SCXRD while the remaining crystallisations which all contained powder were uncapped and the solvents were left to evaporate off at room temperature. The resulting dry powders were transferred to a PXRD plate and analysed before being placed in a vacuum oven at 80 °C overnight before the PXRD patterns were recollected.

Table S4. The concentrations of the TSPM and TAPM stock solutions that were used during the HT crystallisations, along with the amounts used.

TSPM			TAPM		
**Solvent code**	**Solubility (mg/ml)**	**Conc. Used (mg/ml)**	**Solvent code**	**Solubility (mg/ml)**	**Conc. Used (mg/ml)**
10	2.5	2	10	6	2.5
11	1.6	1	12	15	2.5
14	10	2	15	15	2.5
15	1	1	18	5	2.5
19	2.5	1	20	5	2.5
21	15	2	26	1.25	1
23	1.6	1	30	15	2.5
24	5	2	35	15	2.5
27	10	2			
29	15	2			
30	2	1			
35	15	2			
37	15	2			

The rate of addition of the TAPM stock solutions to the TSPM solutions was varied, the rate of 1.0 ml/min gave mainly semi-amorphous materials. When the rate of addition was decreased down to 0.1 ml/min the crystallinity of many of the conditions were drastically improved (**Figure S5**). The crystallisation screen gave many different PXRD patterns. Those that occurred in 10 or more conditions were grouped into phases. These phases are highlighted in **Figure 2c** and their PXRD patterns can be seen in **Figure 2b**.

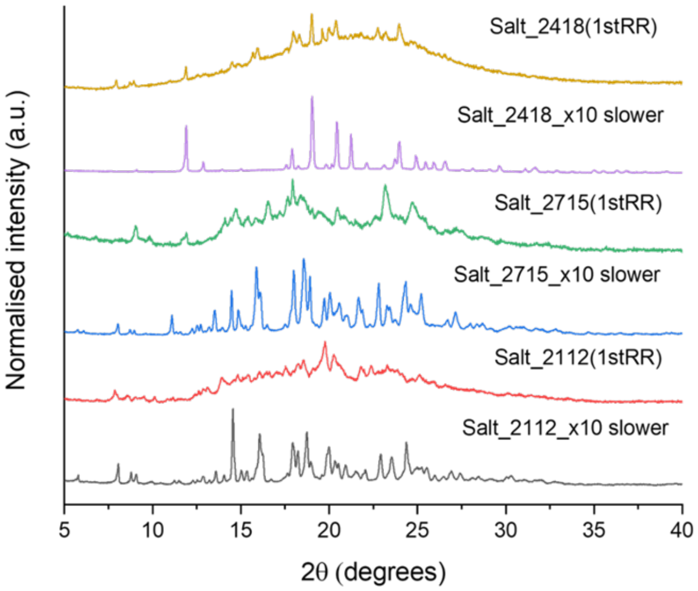

Figure S5. PXRD patterns for some of the crystallisation samples comparing the crystallinity when the rate of addition of the TAPM to the TSPM was varied.

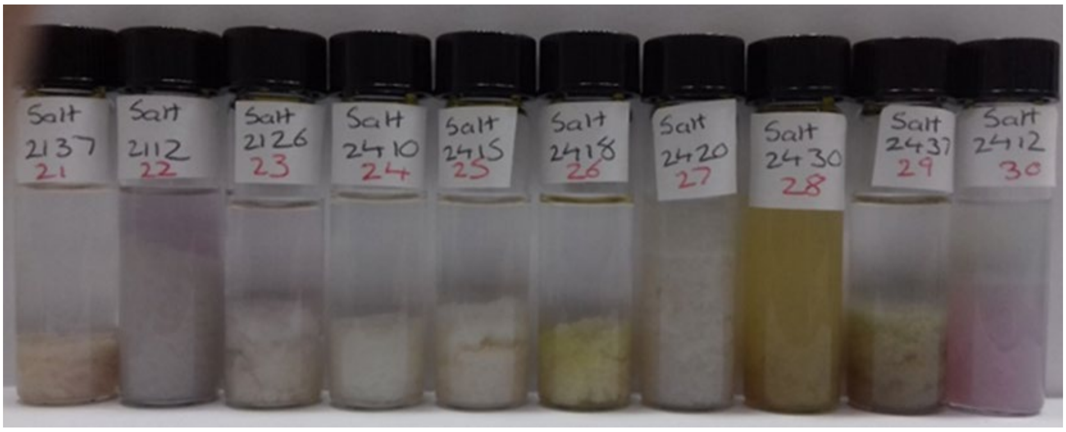

Figure S6. Images of the vials containing the salt crystallisation immediately after taking the samples off the robot. Precipitation was observed in all robot runs irrespective of the rate of addition (except for the crystallisations using water).

CPOS-7 crystal structure

Single crystals of **CPOS-7** were grown in a number of solvent conditions during the HT crystallisation screen. The crystal data collected from EtOH/Dioxane is discussed here; the structure contained diffuse electron density within the channels which could not be modelled as the solvents, therefore solvent mask was used (estimated total electron count: 316, estimated total volume: 822 Å^3). Due to disorder in the structure a number of restraints and constraints were used for modelling including

RIGU, SADI, DFIX, DELU and SIMU. For a displacement ellipsoid plot of the asymmetric unit cell see **Figure S7**.

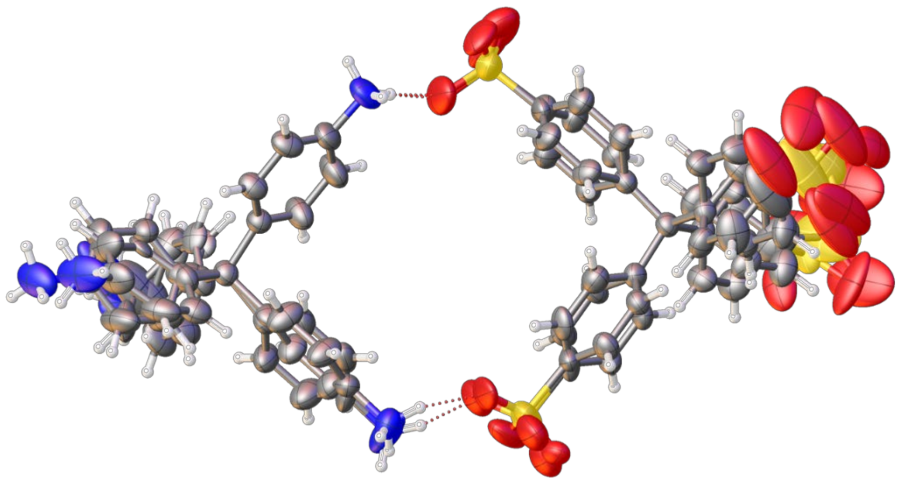

Figure S7. a) Displacement ellipsoid plot from the single crystal structure of **CPOS-7**. Ellipsoids are displayed at 50% probability. Atom labels are omitted for clarity. Grey: carbon; Red: oxygen; yellow: sulphur; blue: nitrogen; white: hydrogen.

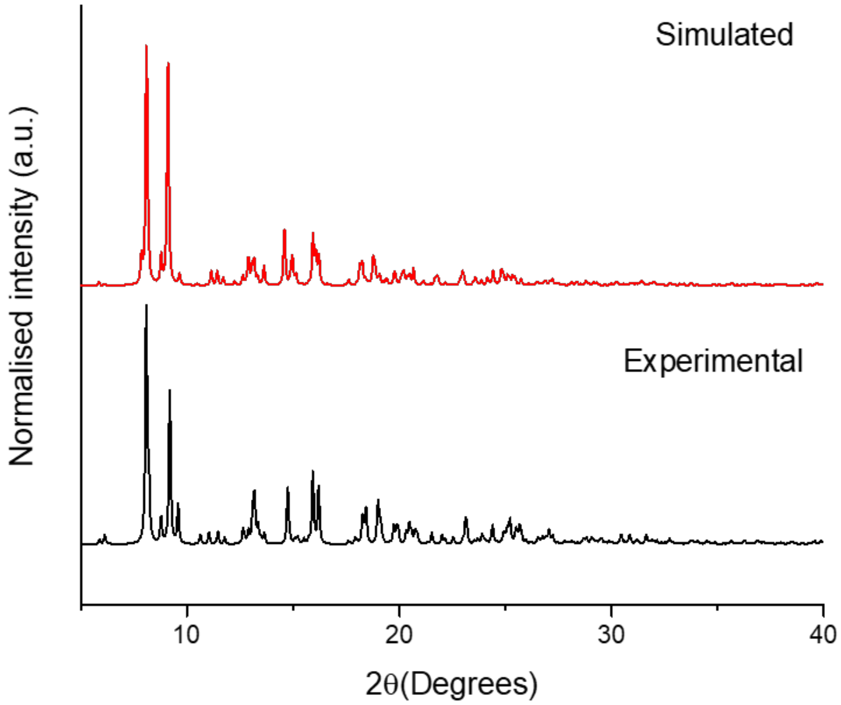

Figure S8. PXRD comparison of the simulated PXRD pattern from the single crystal structure of **CPOS-7** grown from EtOH/Dioxane and the experimental sample which has been prepared by drying and grinding to give a fine powder. Throughout the rest of the SI and the manuscript unless stated all PXRD samples were measured without any drying or grinding prior to the measurement.

Hydrate2920 crystal structure

Single crystals of **hydrate2920** were grown during the HT screen in several water conditions, but the data presented here is from water/trifluoroethanol. Crystal data for **Hydrate2920**: Formula $C_{25}H_{16}O_{12}S_4$·8($H_2$0); M = 1165.26, Tetragonal *I*41/a, colourless needle shaped crystals; crystal size = 0.28 x 0.07 x 0.03 mm^3; a = 19.4128(3) Å, b = 19.4128(3) Å, c = 14.1983(11) Å, α,β,γ, = 90, V = 5356.0(2) $Å^3$; ρ = 1.445 g cm^{-3}; μ(rotating anode Mo-Kα λ = 0.71073 Å) = 0.259 mm^{-3}; *F* (000) = 2448; T = 200 K; 42074 reflections measured (3.552 < 2Θ < 65.774 °), 4870 unique (R_{int} = 0.0716), 3243 ($I > 2\sigma(I)$); R_1 = 0.0729 for observed and R_1 = 0.1210 for all reflections; wR_2 = 0.1738 for all reflections; max/min difference electron density = 0.416 and -0.340 e.A^{-3}; data/restraints/parameters = 4870 / 0 / 255; GOF = 1.061. See **Figure S9a** for a displacement ellipsoid plot of the asymmetric unit cell. For the packing along the *b,c,d* axes see **Figure S9 b,c,d**.

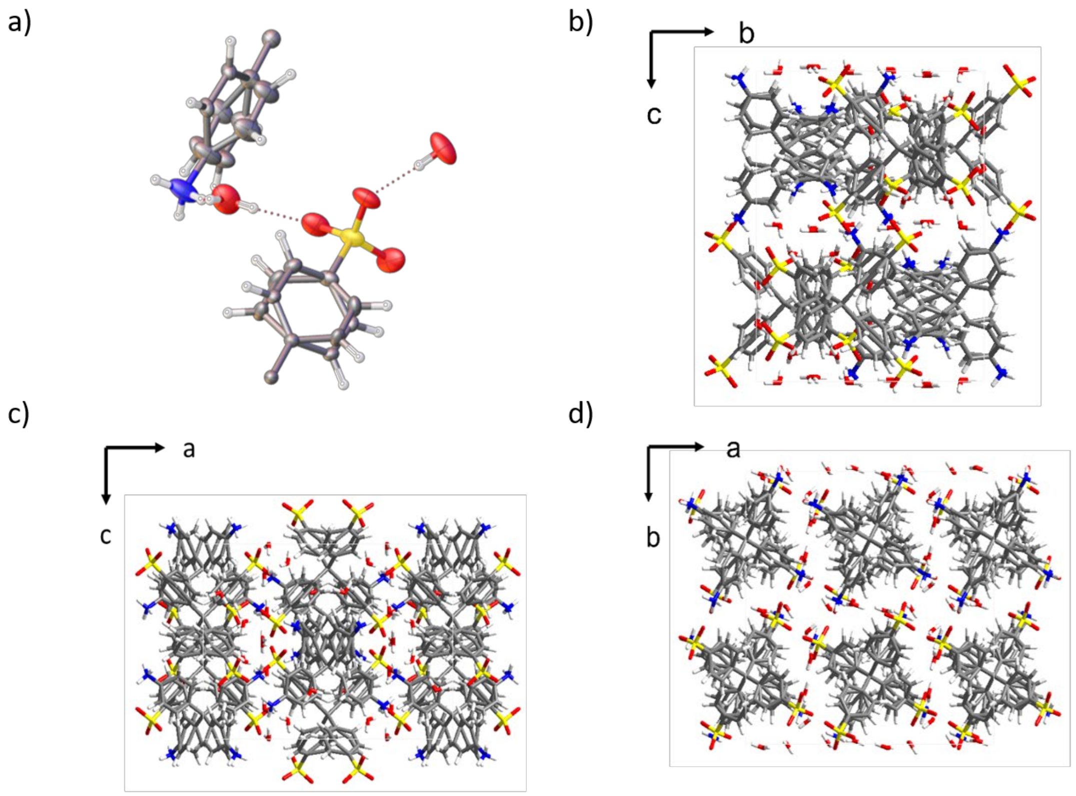

Figure S9. a) Displacement ellipsoid plot from the single crystal structure of **Hydrate2920**. Ellipsoids are displayed at 50% probability. The disordered carbon atoms of the benzene rings are modelled with 50% occupancy. Atom labels are omitted for clarity. b,c,d) Crystal packing of **Hydrate2920** viewed along the *a*, *b* and *c* crystallography axis respectively. Grey: carbon; Red: oxygen; yellow: sulphur; blue: nitrogen; white: hydrogen.

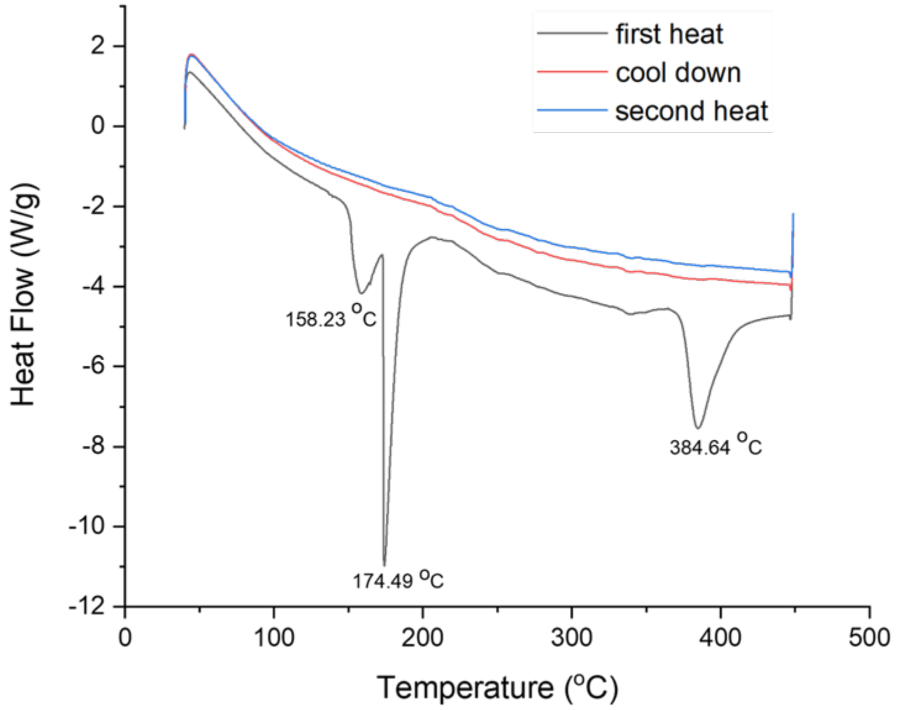

Figure S10. DSC plot for **Hydrate2920** recorded over temperature range 40 °C to 450 °C, using heat-cool-heat cycle procedure. Black: first heating procedure from 40 °C to 450 °C; red: cooling from 450 °C to 40 °C; blue: second heating procedure from 40 °C to 450 °C.

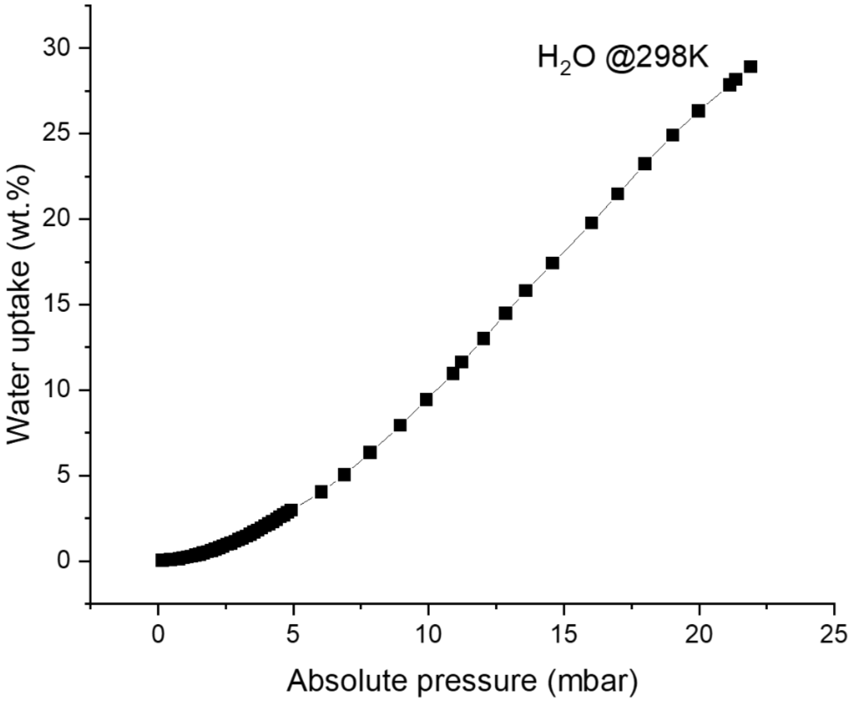

Figure S11. Water isotherm for **Hydrate2920** at 298 K after heating under vacuum at 150 ^{o}C overnight, showing a wt.% uptake of 30%.

Scaling of CPOS-7 in batch

Many attempts were made at scaling **CPOS-7** in batch focusing on the conditions using EtOH/dioxane and THF. The main outcomes from the scale up attempts were either amorphous, **Hydrate2920** or mixtures of **CPOS-7** and **Hydrate2920**. The PXRD patterns from the scale-up attempts can be seen in **Figure S12** and a summary of the outcomes of the reactions can be seen in **Table S5**. During one attempt where the reaction was done under inert conditions using anhydrous EtOH/dioxane with the TSPM solution (EtOH) heated at 80 ^{o}C, CPOS-7 was scaled to 50 mg (**Table S5, entry 9**).

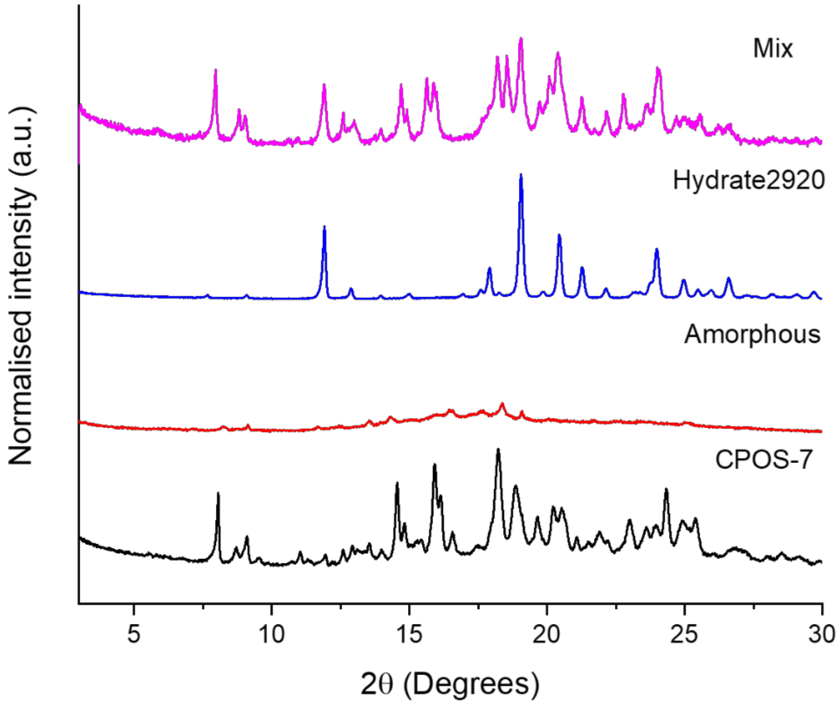

Figure S12. PXRD patterns for the main outcomes of the many attempts at scaling **CPOS-7** in batch using EtOH/Dioxane as the chosen crystallisation solvents.

Table S5. Scale-up attempts of making **CPOS-7** in batch.

Scale (mg)	Solvent system	Rate of mixing (mg/min)	Condition	Outcome
40	THF	0.5	Air	Amorphous
80	THF	0.5	Air	Amorphous
150	THF	0.5	Air	Amorphous
80	THF	0.1	Air	~10% **CPOS-7**
40	EtOH/dioxane	0.1	Air	Mix
40	EtOH/dioxane	0.5	Air	Mix
80	EtOH/dioxane	0.1	Air	Hydrate
150	EtOH/dioxane	0.1	Air	Hydrate
50	EtOH/dioxane	0.1	N_2	**CPOS-7**

Solving the Hydrate issue

It was found that by using sufficient amounts of acetic acid (GAA) prevented the formation of **Hydrate2920** without the need for inert conditions. Using scales of 12.5 mg with 4 mL of EtOH and 2 mL of dioxane, **Hydrate2920** formed when 0.1 mL of GAA was added to the TSPM solution. Increasing the volume of GAA to 0.5 mL and 1 mL prevented the formation of **Hydrate2920** and **CPOS-7** was obtained (**Figure S13**).

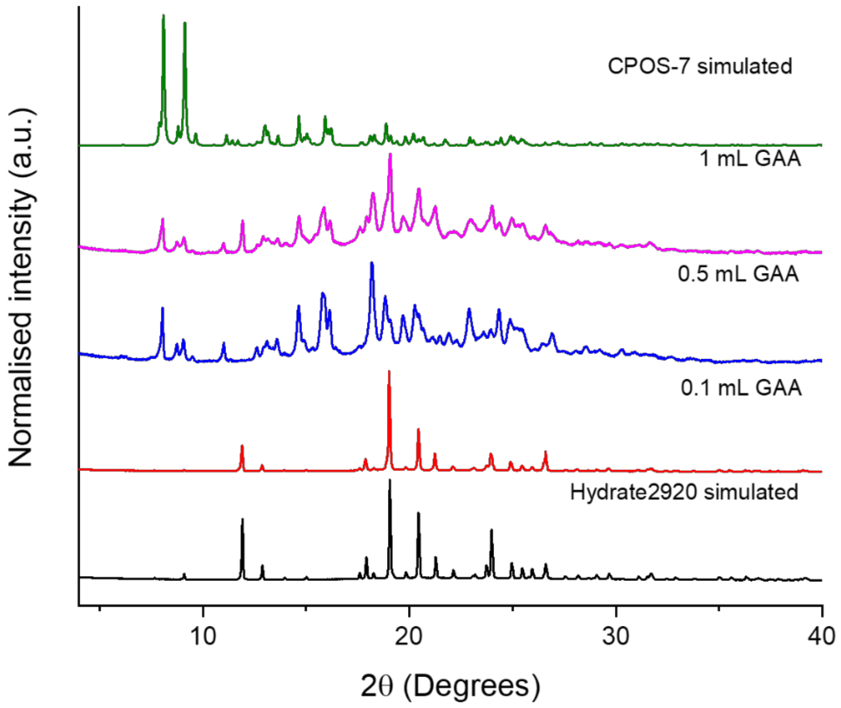

Figure S13. PXRD patterns collected from the tests reactions using various amounts of GAA when trying to scale **CPOS-7** at a rate of 0.5 mg/mL. The results show that with the addition of 0.5 mL and 1 mL of GAA, **CPOS-7** is formed, whereas at lower additions of GAA (0.1 mL) **Hydrate2920** is formed.

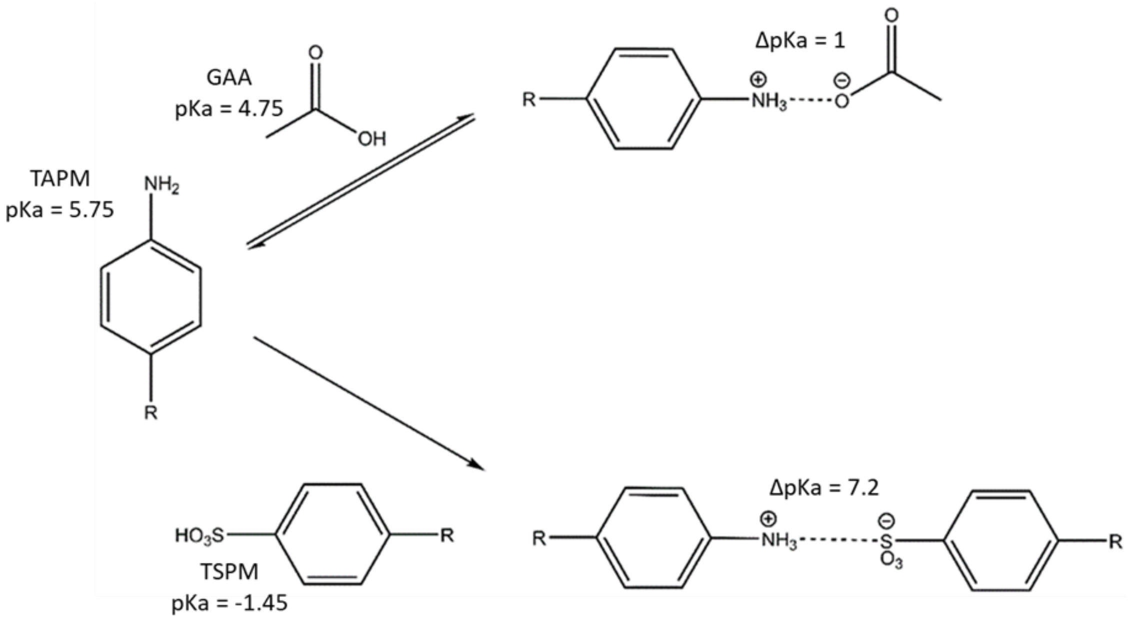

Figure S14. Schematic representation of the potential reaction of acetic acid (GAA) with TAPM to form a salt complex. The pka's of GAA, TSPM and TAPM are included along with the ΔpKa of the resulting acetic acid salt (1) with a comparison to the ΔpKa of CPOS-7 (7.2). The difference between the ΔpKa of the acetic acid salt and that of **CPOS-7** shows the potential for the acetic acid salt to be reversible owing to the much weaker interaction, meanwhile the formation of **CPOS-7** is irreversible during the crystallisation as a consequence of the strength of the interaction.

With the success at preventing **Hydrate2920** from forming, the use of GAA was tested to see if Hydrate2920 and amorphous material could be converted to **CPOS-7**. The results of the tests (Table S6) showed that in cases when starting with the amorphous salt the material never changed as shown by PXRD (**Figure S15**). However, when starting with **Hydrate2920** and using temperature of > 50 ^{o}C it was observed that the material was converted to **CPOS-7** (**Table S6, entry 1**). During these reactions there was no clear dissolution of the material, and the resulting **CPOS-7** was shown to have high crystallinity and recovered in quantitate yields. It should be noted that while there was no observed dissolving of **Hydrate2920**, due to the instant precipitation of **CPOS-7** it is hard to say for certainty if the reaction occurs without the material dissolving in at least very small quantities.

Table S6. Table showing the results of the tests to form **CPOS-7** using GAA from the amorphous samples and **Hydrate2920**.

STARTING MATERIAL	TEMPERATURE (°C)	TIME	GAA ADDED	RESULT
Hydrate2920	50 - 60	24	Yes	**CPOS-7**
Hydrate2920	50 - 60	24	No	**Hydrate2920**
Hydrate2920	RT	24	No	**Hydrate2920**
Hydrate2920	RT	24	Yes	Mixture
Amorphous salt	50 - 60	24	Yes	Amorphous
Amorphous salt	50 - 60	24	No	Amorphous
Amorphous salt	RT	24	No	Amorphous
Amorphous salt	RT	24	Yes	Amorphous

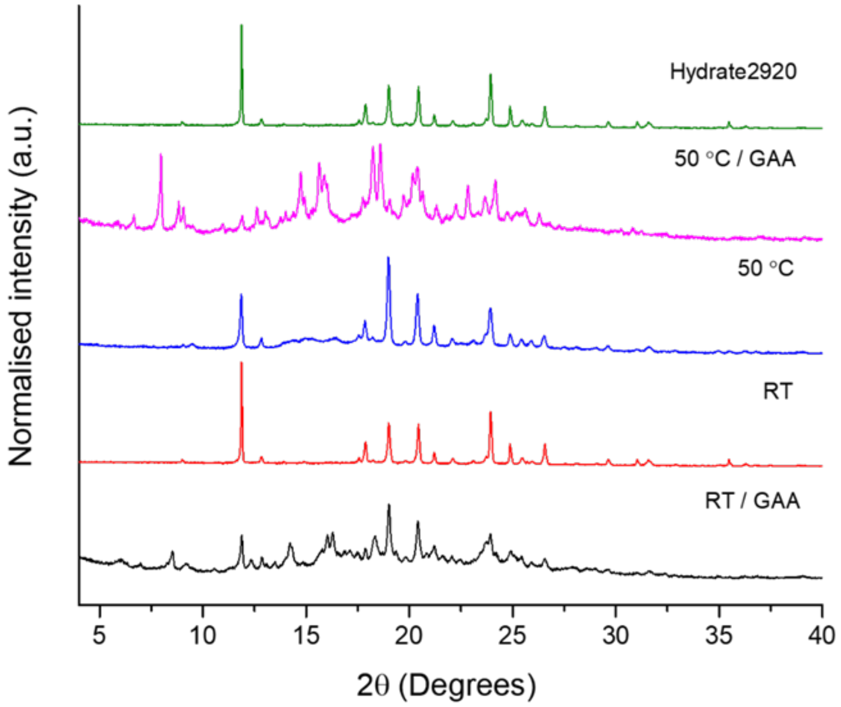

Figure S15. PXRD patterns for samples of **Hydrate2920** after various tests to see the effects. The details of each can be seen in the labels. In all samples, the solvents used was EtOH/Dioxane, for those where GAA was added the amount used was 0.5 mL.

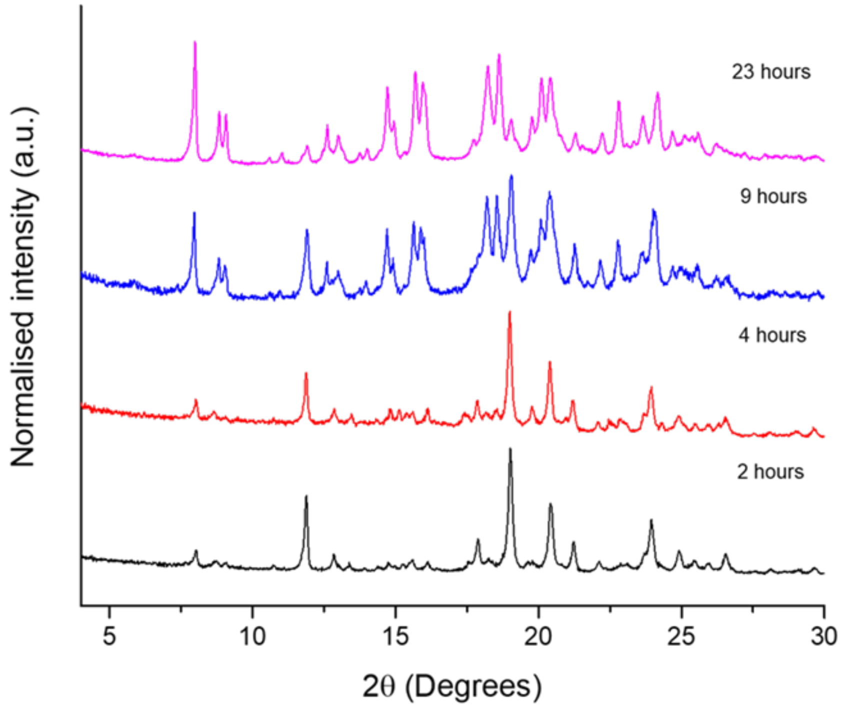

Figure S16. PXRD results from aliquots of a reaction where 12.5 mg of **Hydrate2920** was stirred in EtOH/Dioxane/GAA (4 ml/2 ml/0.5 ml) at 50 °C for 23 hours. The results show that as the reaction proceeds the peaks for **CPOS-7** increase as the **Hydrate2920** decreases.

Stability and activation of CPOS-7

To activate **CPOS-7**, it was held under high vacuum at 150 °C for 16 hours. A TGA plot of the sample after activation recorded under a dry nitrogen flow showed that **CPOS-7** lost ~1.8% mass loss before 100 °C (**Figure S17**) which is most likely due to physisorbed water. The next mass loss in the TGA plot is at ~300 °C. The TGA results indicate that the material is fully activated (**Figure S17**). To check the stability of **CPOS-7** to activation PXRD was recorded after the activation method. The PXRD showed that the structure had remained stable through the removal of the guest from the pores, with no decrease in crystallinity (**Figure S18**).

CPOS-7 is predicted to have a contact surface area of 974.35 Å which makes up 31.0 % of the unit cell volume (**Figure S19**). CO_2 and N_2 gas isotherms were collected for **CPOS-7** to test its permeant porosity, and the results show that the material selectively adsorbs CO_2 over N_2 (**Figure 4** for CO_2 isotherm and **Figure S20** for N_2 isotherm).

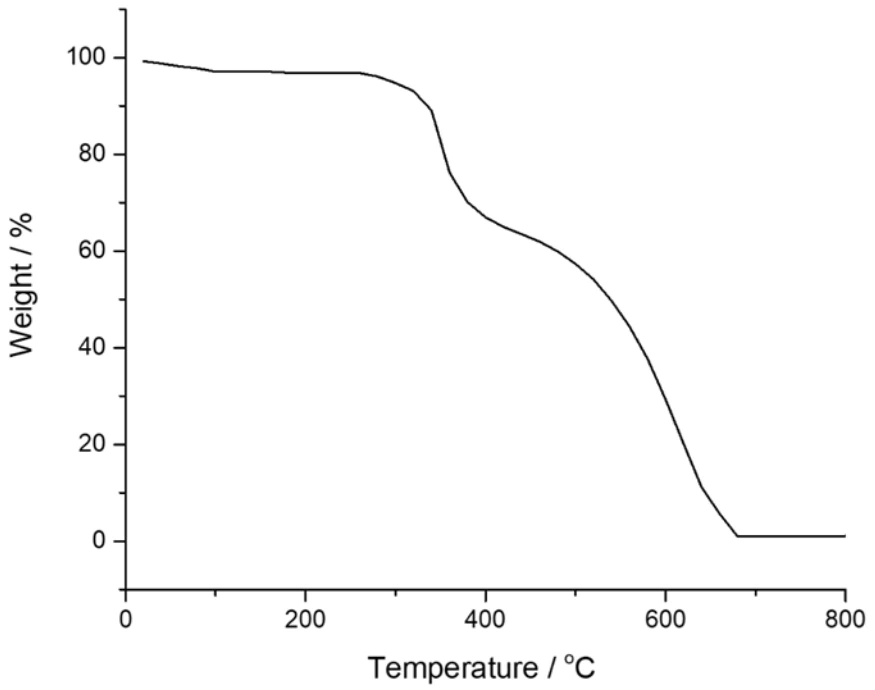

Figure S17. TGA plot of **CPOS-7** reordered under a dry nitrogen gas flow after activation under vacuum at 150 °C for 16 hours.

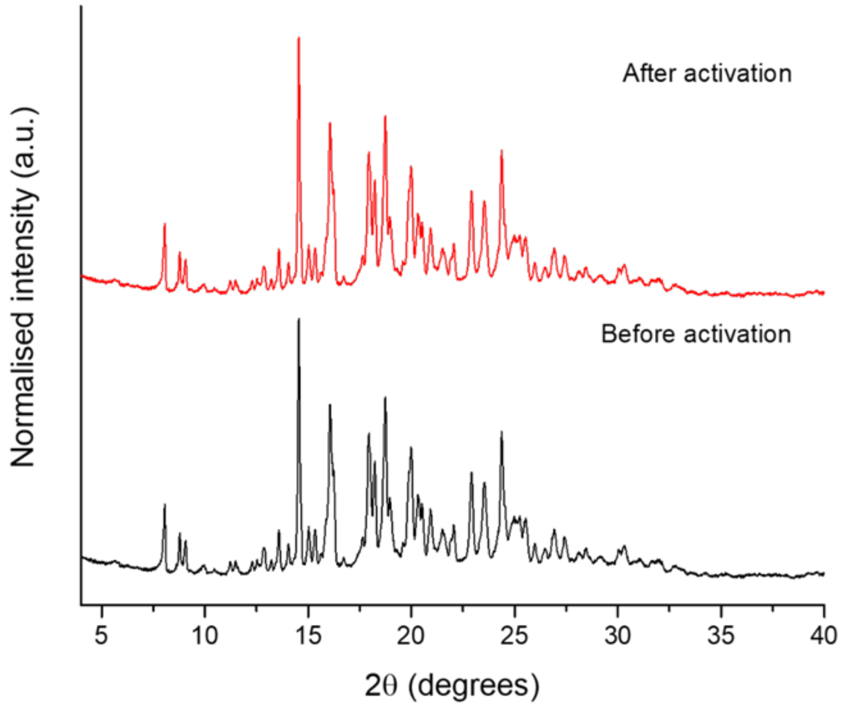

Figure S18. PXRD for sample of **CPOS-7** used for gas isotherm measurements recorded before and after activation at 150 °C under vacuum for 16 hours.

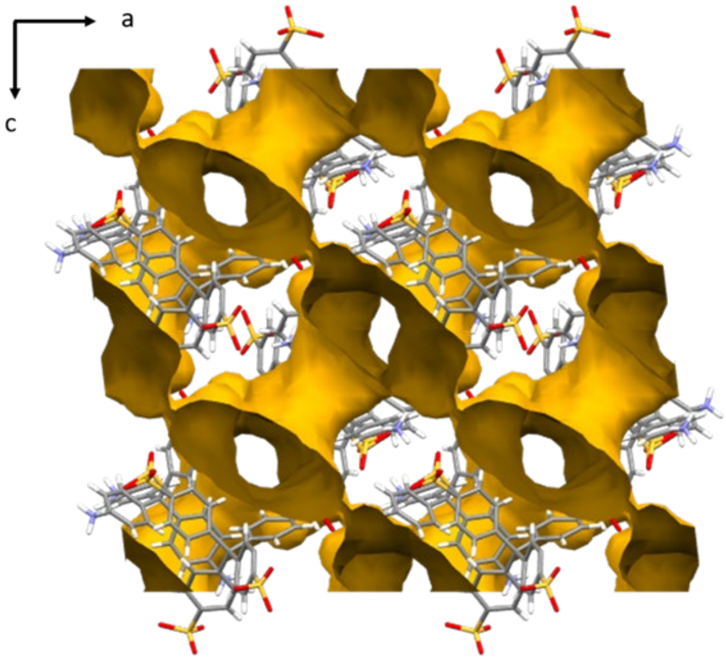

Figure S19. Crystal packing of **CPOS-7** down the *b* axis. Surface contact voids are shown in yellow using a probe radius of 1.2 Å and grid spacing of 0.7 Å.

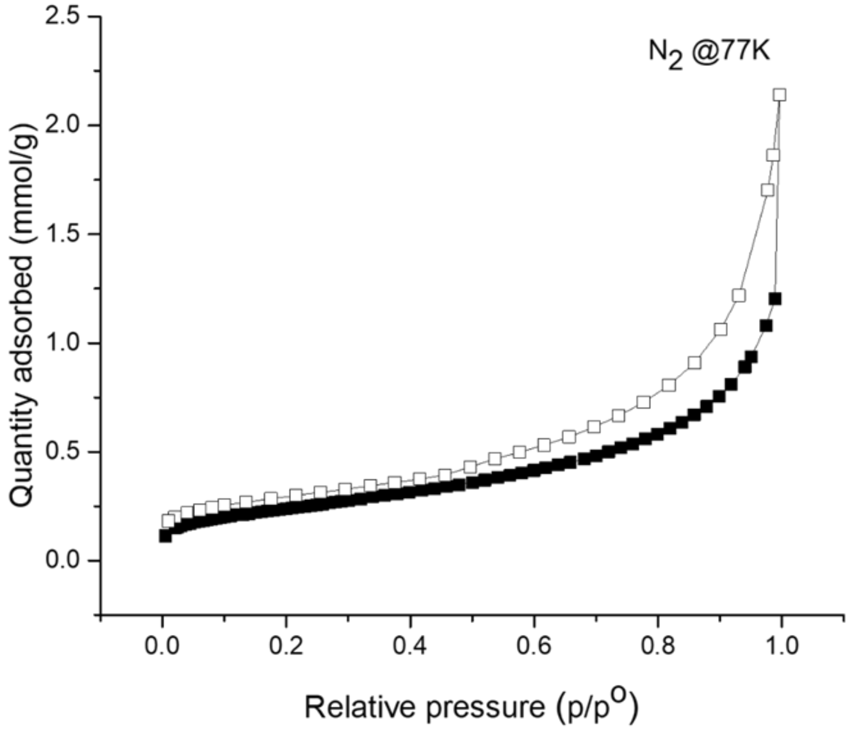

Figure S20. N_2 adsorption/desorption isotherms for **CPOS-7.** Closed symbols are for adsorption points, while open symbols are for desorption points.

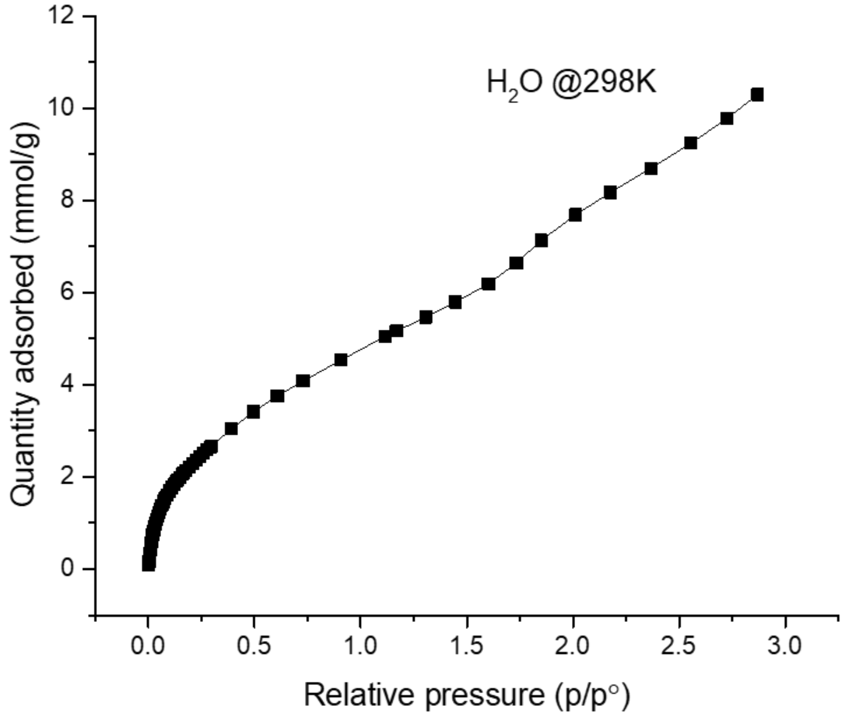

Figure S21. Water isotherm for **CPOS-7**.

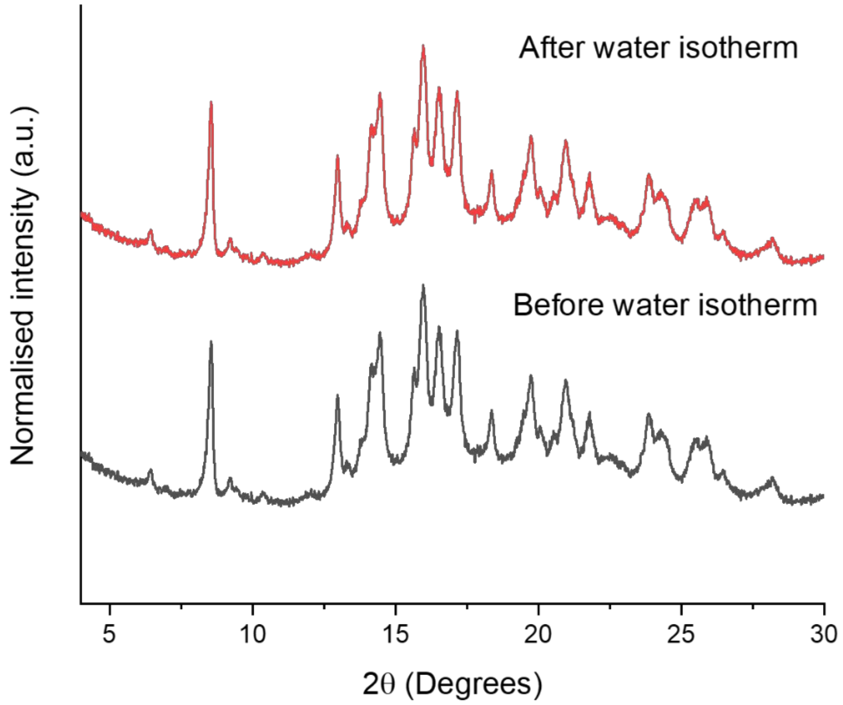

Figure S22. PXRD pattern of the **CPOS-7** sample used for the water isotherm recorded before and after the measurement. There is no indication of any peaks for **Hydrate2920**, showing the material is resistant to water once formed.

Targeting the continuous synthesis of CPOS-7 – Flow Reaction Experimental Details

Initial experiments were carried out with a Harvard Apparatus syringe pump (**Figure S23**), but with no back-pressure regulator used for fear of blocking – a hazard magnified by the lack of pressure regulation of syringe pumps. This was not an issue at room temperature for THF, and the high boiling point of dioxane rendered it unnecessary as well even at 80 °C. The general reaction method for the syringe pump usage is summarised in the schematic shown in **Figure S24**.

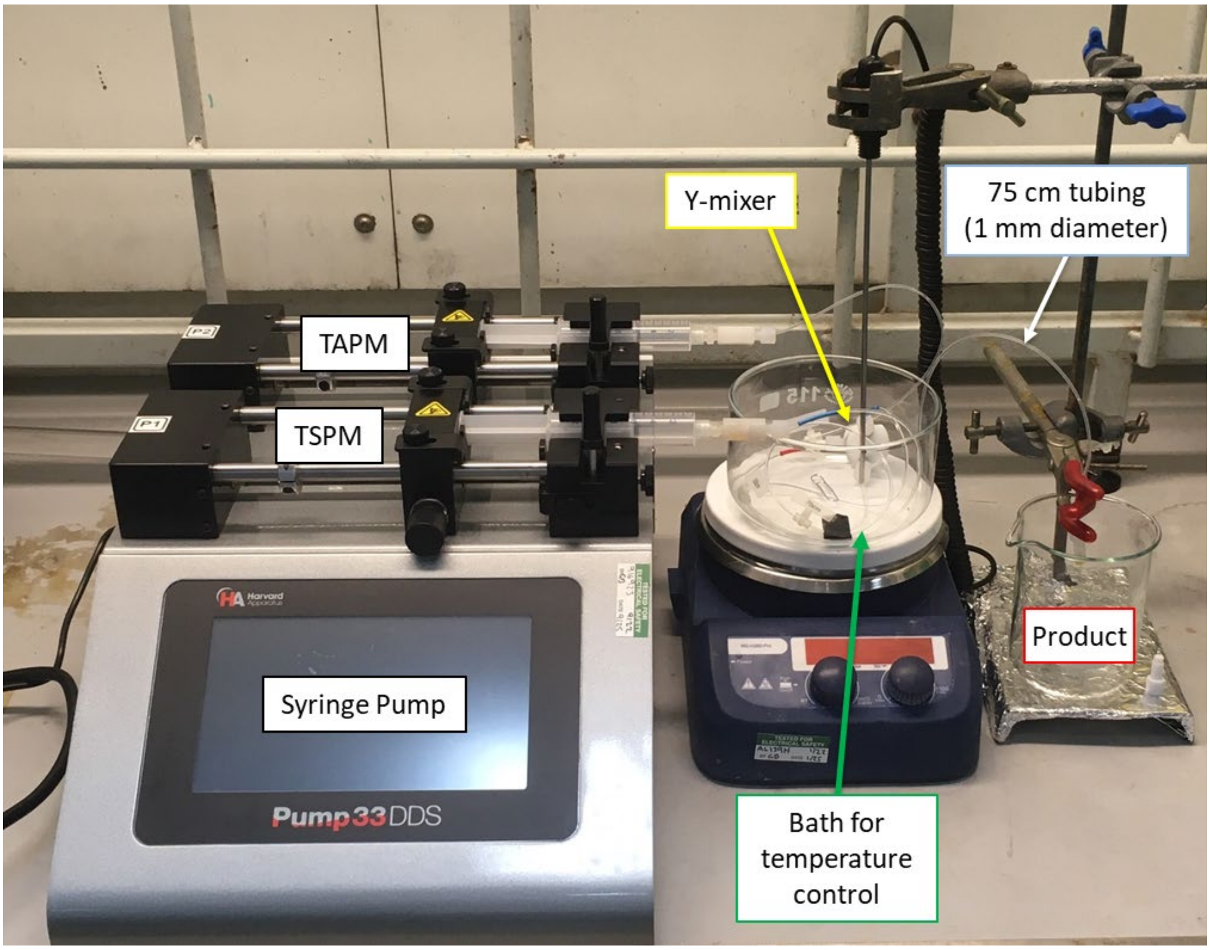

Figure S23. Syringe pump setup

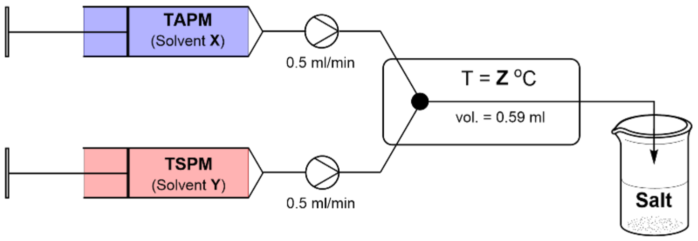

Figure S24. Syringe pump schematic for the flow synthesis of **Salt** products;

Method A: **X =** Dioxane, **Y =** Ethanol, **Z** = 80 °C;

Method B : **X + Y =** THF, **Z =** 20 °C

Experimental Methods

Method A: Solutions of TAPM (0.63 mg/mL) in dioxane and TSPM (1 mg/mL) in ethanol were made, and transferred to syringes, which were then attached to the syringe pump. Both syringes were set to 0.5 mL/min flow rates, and injected into pre-warmed tubing submerged in a water bath set to 80 °C. The two streams combined in a Y-mixer submerge in the water bath, and flow through 0.59 mL of heated tubing before being collected at the outlet into a waste or sample vial. The steady state was collected after 1 minute of reaction throughput, and the sample collected was then concentrated over a few days to yield the product (**Hydrate2920**).

Method B: Solutions of TAPM (0.63 mg/mL) and TSPM (1 mg/mL) in THF were prepared and transferred to syringes, which were attached to the syringe pump. Both syringes were set to 0.5 mL/min flow rates, and injected into tubing at room temperature. The two streams combined in a Y-mixer and flowed through 0.59 mL of tubing before being collected at the outlet into a waste or sample vial. The steady state was collected after 1 minute of reaction throughput, and the desired sample collected was then concentrated over a few days to yield the product (**Hydrate 2920**).

Effect of Flow Rate and Residence Time: Solutions of TAPM (0.63 mg/mL) and TSPM (1 mg/mL) in THF were prepared and transferred to syringes, which were attached to the syringe pump. Both syringes were set to equal flow rates between 0.05 – 1.0 mL/min, and injected into tubing at room temperature. The two streams combined in a Y-mixer and flowed through 0.59 mL of tubing before being collected at the outlet into a waste or sample vial. The steady state was reached after 10% of the reaction throughput, and the desired sample collected was then concentrated over a few days to yield the product (**Hydrate 2920**). See **Figure S23** for the PXRD results of these tests.

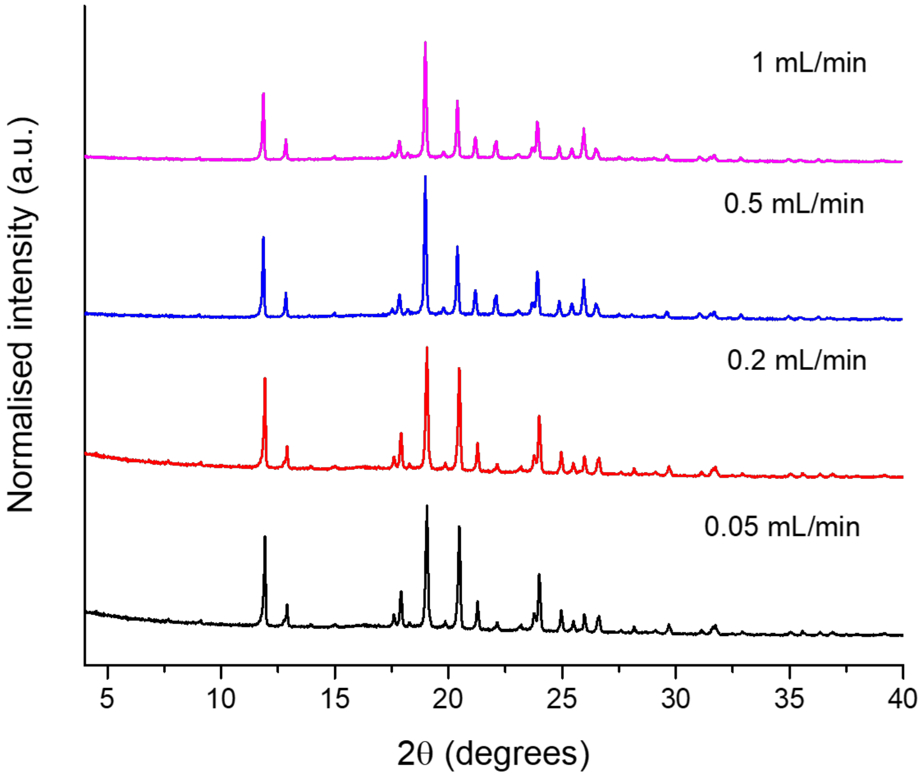

Figure S25. The effect on flow rate highlighted a loss of crystallinity above a total flow rate of 1.0 ml/min, so this was chosen as optimal as it delivered the highest throughput without loss of quality in the product.

Method C: Solutions of TAPM in THF (0.63 mg/ml) and freshly freeze-dried TSPM in 6.7% GAA (v/v) in THF (1 mg/ml) were prepared and transferred to syringes, which were attached to the syringe pump. Both syringes were set to equal flow rates of 0.5 ml/min and injected into tubing at room temperature. The two streams combined in a Y-mixer and flowed through 0.59 ml of tubing before being collected at the outlet into a waste or sample vial. The steady state was reached after 10% of the reaction throughput, and the desired sample was collected. The sample was left to settle over 24 hours, the excess solvent decanted off, and then dried at room temperature to yield the product (**CPOS-7**) (**Figure S26**).

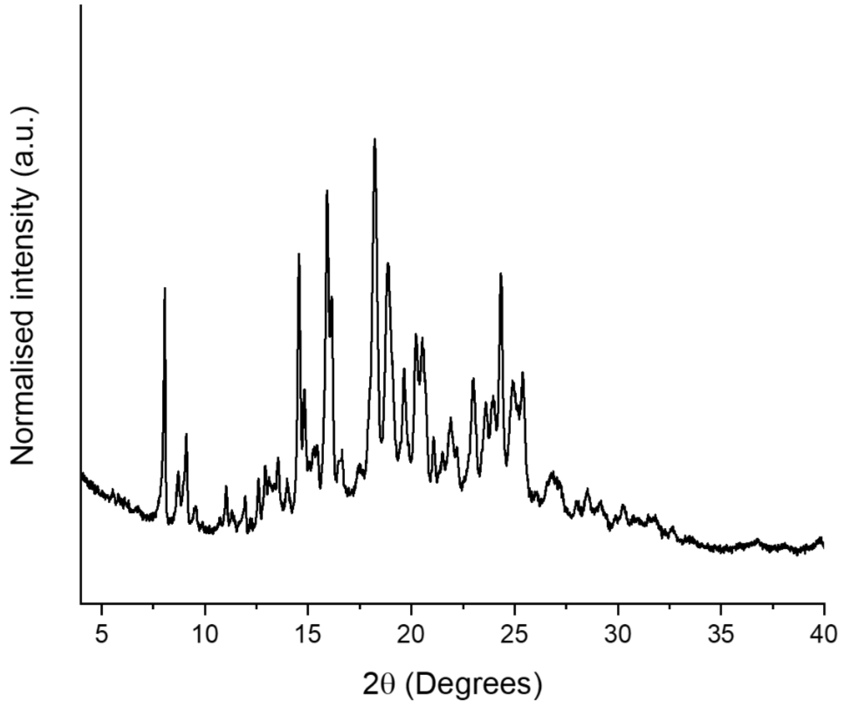

Figure S26. PXRD pattern of the **CPOS-7** formed during method C using flow chemistry.

Scale-up Flow Reaction

Further scale up reactions were carried out using a Vapourtec E-series reactor (**Figure S23**), using two peristaltic pumps to mix the solutions. No reactor coils were needed, with the mixing piece and tubing kept at room temperature with a short residence time. Reactions were run using freshly freeze-dried TSPM (20% H_2O w/w) to ensure the non-hydrated salt was the product.

Method D: 0.0016 mM solutions of TSPM (96 mg (20% H_2O) in 5 ml GAA and 75 ml THF) and TAPM (50 mg in 80 ml THF) were prepared and transferred to conical flasks. The solutions were then pumped at 0.5 ml/min and combined in a Y-mixer, flowing through 0.59 ml of tubing before collecting in a vial. The system was left to reach steady state for 1 minute, then the following 159 ml was collected in a beaker. After letting the solid settle, the excess solvent was decanted off, and the resulting solid left to dry to yield **CPOS-7** (123 mg, 96%)

The **CPOS-7** sample was then measured and gave the PXRD in **Figure 4.**

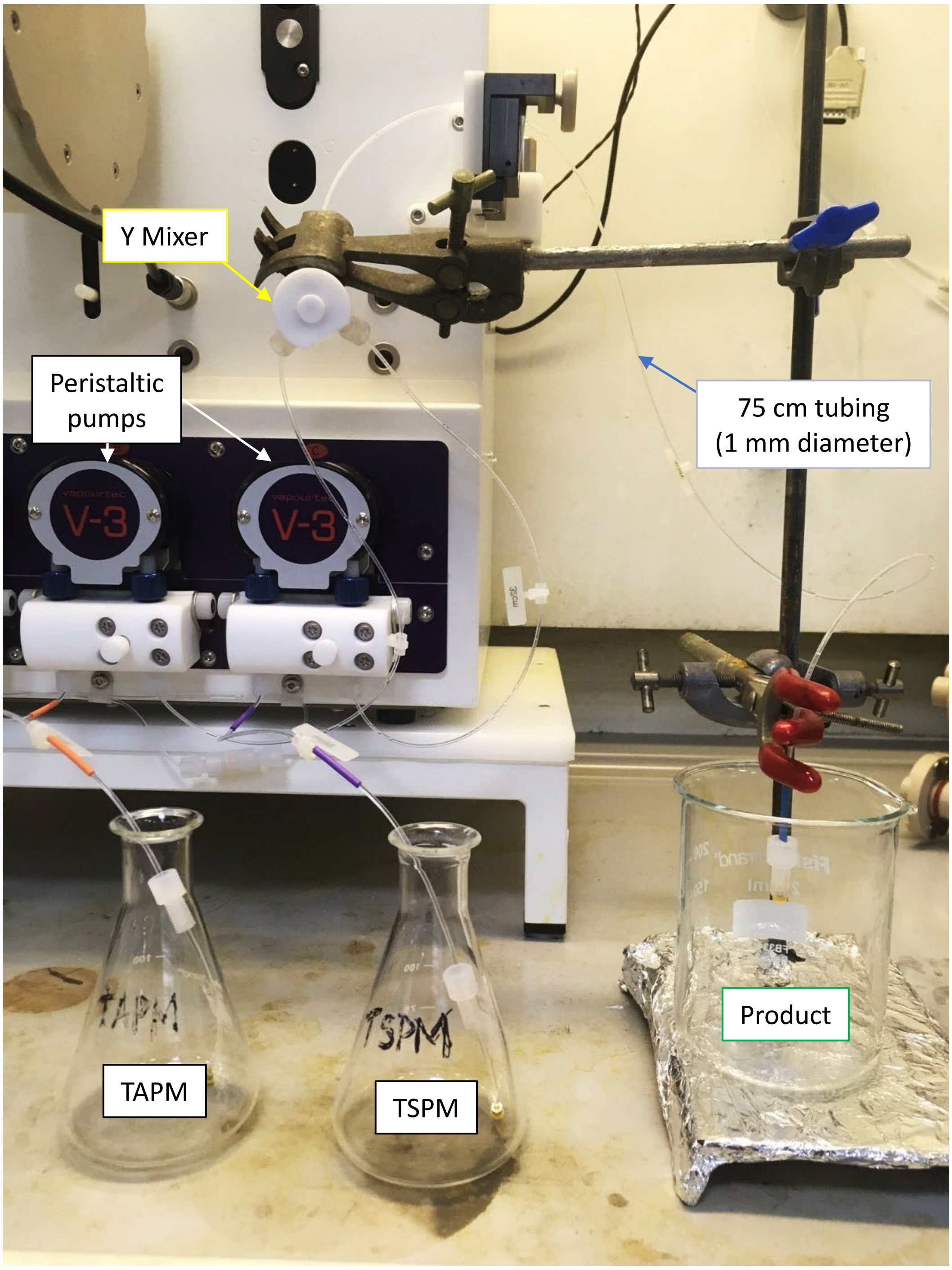

Figure S27. Vapourtec E-series Flow Reactor and reaction setup

Higher Concentration Study

To test that the results in flow were not being affected by the difference in concentration of the TAPM solution (2.5 mg/ml in batch vs 1 mg/ml in flow), the flow reactor test was done using the same concentrations that were used in batch, with the rate of mixing between TSPM and TAPM a 1:2 ratio

on a 25 mg scale. The results showed that even with using the increased concentration of the TAPM solution and the difference in the rate of each solution **CPOS-7** was still formed (**Figure S28**).

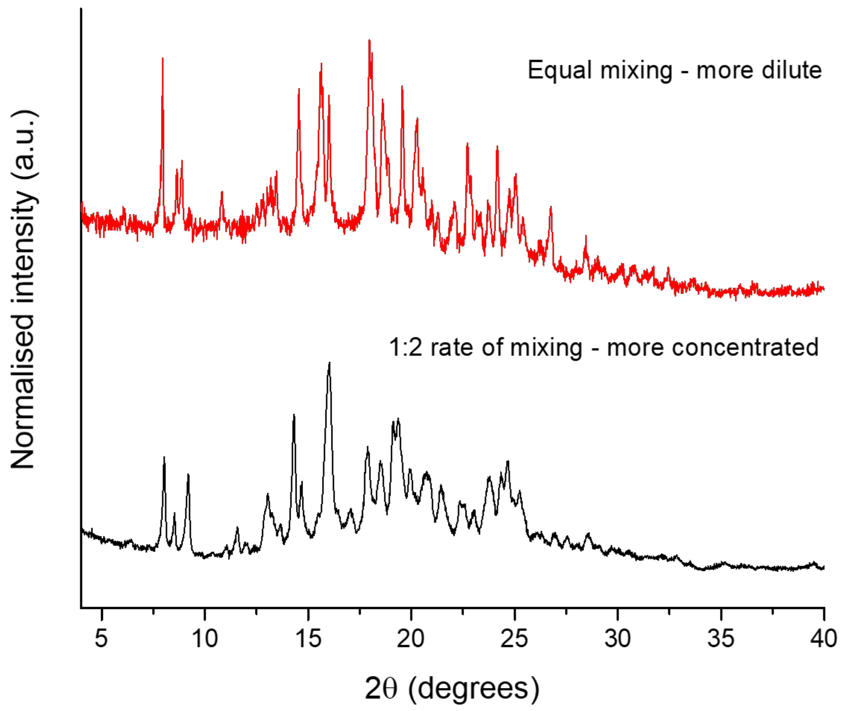

Figure S28. PXRD patterns from crystallisations using the flow reactor at different concentrations, where **CPOS-7** was formed in both cases.

Other Observations

During one isolated test (50 mg scale), the reactor had been used previously using 2-propanol and wasn't fully flushed with THF before the reaction started. The results of this test showed that the steady state sample and the end of the reaction which didn't contain any 2-propanol gave **CPOS-7**; however, the start of the reaction (which was likely contaminated with a very small amount of 2-propanol) gave another polymorph. The PXRD pattern for the polymorph is very similar to one formed during the HTS using conditions butanol/tetrahydropyran (**Figure 2c**). While this result was not intentional, it does show that other polymorphs of the salt can be made in flow, in addition to **Hydrate2920** and **CPOS-7**.

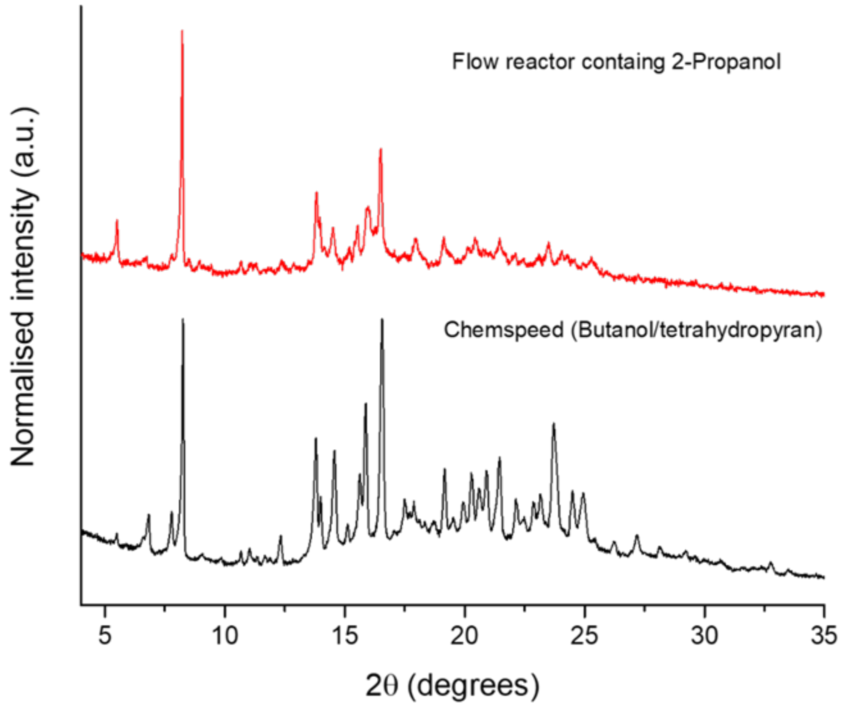

Figure S29. Comparison of the PXRD patterns from the HT screen using butanol/tetrahydropyran and from the flow reactor sample where the reactor was contaminated with 2-propanol.

Confirmation of Flow Sample Porosity

To verify that the flow scale up reactions gave the same quality material as that of batch, gas sorption studies were run on the >100 mg of sample that was obtained with flow. Pleasingly, the results of the studies show that the material made in flow continues to show comparable porosity as that of previous samples, with the only lessened activity being noticed in the 195 K tests (**Figure S30**).

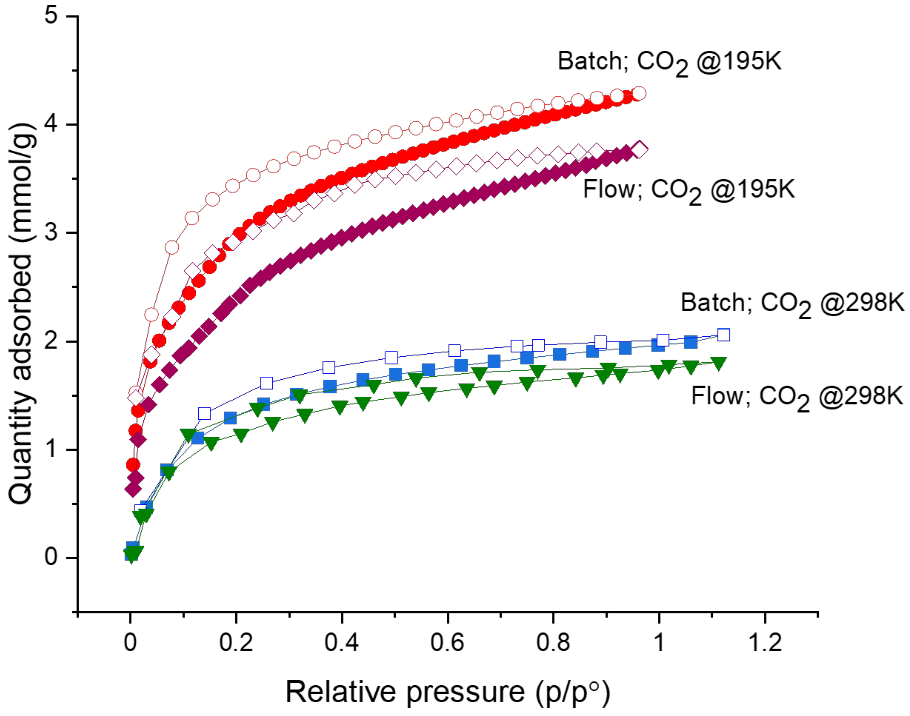

Figure S30. CO_2 isotherms for **CPOS-7** made using flow (flow) and from the reconstruction (batch). Filled symbols are for adsorption and empty is for desorptions.

Crystallography data tables

Table S7. Crystallography tables for **TAPM_19, TSPM_37** and **TAPM_2610**.

Molecule	**TSPM_19**	**TSPM_37**	**TAPM_2610**
λ [Å]	0.71073	1.54178	0.6889
Collection Temperature	100 K	100 K	100 K
Formula	$C_{25}H_{20}O_{12}S_4$·4(H_20) ·2($C_4H_8O_2$)	$C_{25}H_{20}O_{12}S_4$·4.7(C_5H_8 O) ·8.25(H_20)	$C_{25}H_{24}N_4$·$C_5H_{10}O$
Mr [g mol-1]	721.71	1184.61	466.61
Crystal Size [mm]	0.08 x 0.02 x 0.02	0.37 x 0.36 x 0.35	0.06 x 0.06 x 0.02
Crystal System	Monoclinic	Tetragonal	Triclinic
Space Group	P2/*n*	*P4/n*	$P\overline{1}$
a [Å]	16.592(4)	19.2390(14)	10.66710(5)
b [Å]	7.3530(17)	19.2390(14)	11.18920(5)
c [Å]	16.844(4)	7.6186(5)	12.31930(5)
A [°]			116.3370(4)
β [°]	97.743(6)	90	99.4340(4)
γ [°]			96.3190(4)
V [Å^3]	2036.3(8)	2819.9(5)	1271.850(10)
Z	2	2	2
D_{calcd} [g cm^{-3}]	1.450	1.395	1.218
μ [mm^{-1}]	0.313	2.261	0.070
F(000)	9323	1257	500
2θ range [°]	3.704 – 43.934	6.0 – 58.4	3.682 – 56.306
Reflections collected	17118	30588	20344
Independent reflections, R_{int}	2492, 0.1253	2883, 0.0609	6766, 0.0475
Obs. Data [$I > 2\sigma(I)$]	1603	2528	6336
Data /restraints / parameters	2492 / 2 / 241	2883 / 57 / 185	6766 / 0 / 348
Final R1 values ($I > 2\sigma(I)$)	0.0612	0.0840	0.0501
Final R1 values (all data)	0.0985	0.0925	0.0523
Final wR(F_2) values (all data)	0.1692	0.1906	0.1390
Goodness-of-fit on F^2	1.048	1.083	1.021
Largest difference peak and hole [e.A^{-3}]	0.378 / -0.481	0.870 / -0.469	0.524 / -0.306
CCDC	2267604	2267605	2267603

Table S8. Crystallography tables for **CPOS-7** and **Hydrate2920**.

Molecule	**CPOS-7**	**Hydrate2920**
λ [Å]	0.71073	0.71073
Collection Temperature	100 K	200 K
Formula	$C_{25} H_{16} O_{12} S_4$, $C_{25} H_{27.65} N_4$	$C_{25}H_{16}O_{12}S_4$·8($H_2$0)
Mr [g mol-1]	1020.78	1165.26
Crystal Size [mm]	0.05 x 0.048 x 0.043	0.28 x 0.07 x 0.03
Crystal System	Triclinic	Tetragonal
Space Group	$P\bar{1}$	$I4_1/a$
a [Å]	14.4171(6)	19.4128(3)
b [Å]	14.8323(6)	19.4128(3)
c [Å]	15.3011(5)	14.2122(3)
	84.069(3)	
β [°]	82.147(3)	90
	77.676(4)	
V [Å^3]	3157.3(2)	5356.0(2)
Z	2	4
D_{calcd} [g cm^{-3}]	1.074	1.445
μ [mm^{-1}]	0.203	0.259
F(000)	1063	2448
θ range [°]	1.810 – 24.711	3.552 – 65.774
Reflections collected	37190	42074
Independent reflections, R_{int}	10708,0.0309	4870, 0.0716
Obs. Data [I > 2σ(I)]	7998	3243
Data /restraints / parameters	10708 / 1135 / 1022	4870 / 0 / 255
Final R1 values (I > 2σ(I))	0.1380	0.0729
Final R1 values (all data)	0.1581	0.1210
Final wR(F_2) values (all data)	0.3675	0.1738
Goodness-of-fit on F^2	0.838	1.061
Largest difference peak and hole [e.A^{-3}]	1.719 / -0.553	0.416 / -0.340
CCDC	2267601	2267602

Miscellaneous Information

Table S9. Comparison of the best results of scaling **CPOS-7** in batch and flow.

Condition	Scale (mg)	Solvent system	Rate of mixing	Reproducible?
**Batch**	50	EtOH/Dioxane	0.1	No
**Hydrate conversion**	150	EtOH/Dioxane or THF	N/A	Yes
**Flow + GAA**	150	THF	0.5	Yes

References

[1] S. Parsons, ECLIPSE 2004, *Univ. Edinburgh, Edinburgh, UK*.

[2] G. Sheldrick, SADABS, *University of Göttingen, Germany*, **2008**.

[3] G. Sheldrick, *Acta Cryst*. **2015**, A17, 3.

[4] B. Sarma and A. Nangia, *CrystEngComm*, **2007**, 9, 628–631.

[5] A. J. Cruz-Cabeza, *CrystEngComm*, **2012**, 14, 6362–6365.

[6] S. L. Childs, G. P. Stahly, A. Park, *Mol. Pharm.*,**2007**, 4, 3, 323–338.
